# Supplementary material for: Combining cross-sectional and longitudinal genomic approaches to identify determinants of cognitive and physical decline
Source: Nat Commun. 2025 May 15;16:4524. doi: 10.1038/s41467-025-59383-0 (PMC12081661; doi:10.1038/s41467-025-59383-0)
Supplement: Supplementary file 1 — Supplementary Information [file 41467_2025_59383_MOESM1_ESM.pdf]

Supplement File

Contents

Supplementary Methods

Cognitive and physical indexes of age-related decline . . . .

Evaluation of two-wave models of change to study longitudinal genetic effects . . . .

Sample representativeness and correction for selective participation . . . .

Supplementary Discussion

Slopes obtained from three-wave data . . . .

Sample representativeness and correction for selective participation . . . .

Evaluation of two-wave models of change to study longitudinal genetic effects . . . .

Reserach in context . . . .

Supplementary Figures

Supplementary Figure 1. Age effects across measures of physical and cognitive function . . . .

Supplementary Figure 2. Box and scatter plot of cognitive measures included in genome-wide scans

Supplementary Figure 3. Box and scatter plot of physical measures included in genome-wide scans .

Supplementary Figure 4. Correlations between change scores indexing cognitive and physical decline

Supplementary Figure 5. Correlations between baseline scores of cognitive and physical measures . .

Supplementary Figure 6. Evaluation of two-wave models of change for longitudinal genetic effect estimation . . . .

Supplementary Figure 7. Phenotypic predictors of change in the UK Biobank . . . .

Supplementary Figure 8. Longitudinal and cross-sectional genetic effects on cognitive and physical function . . . .

Supplementary Figure 9. Genotype-phenotype associations of variants associated with cognitive or physical decline . . . .

Supplementary Figure 10. Genotype-phenotype associations of variants showing sex-differential effects on physical function (composite score) . . . .
Supplementary Figure 11. Weighted genome-wide analyses of variants associated with physical and cognitive outcomes . . . .
Supplementary Figure 12. Predictors of decline obtained from phenotypic association tests and Mendelian Randomization analyses . . . .
Supplementary Figure 13. Predictors of cognitive decline (Mendelian Randomization) . . . .
Supplementary Figure 14. Predictors of physical decline (Mendelian Randomization) . . . .
Supplementary Figure 15. Longitudinal assessments of aging phenotypes in the UK Biobank . . . .
Supplementary Figure 16. Correlations across measurement occasions for physical measures . . . . .
Supplementary Figure 17. Correlations across measurement occasions for cognitive measures . . . .
Supplementary Figure 18. Flow Diagram of the sample selection process . . . .
Supplementary Figure 19. UK Biobank representativeness and characteristics of selective participation
Supplementary Figure 20. Genome-wide tests on cognitive and physical measures in the UK Biobank follow-up sample and dropout sample . . . .
Supplementary Figure 21. Distribution of baseline and follow—up participation weight . . . .

Supplementary References

Supplementary Methods

Cognitive and physical indexes of age-related decline

For the cognitive measures, most tests were first introduced at baseline in form of a 15-minute computerized battery, where brief and biobank-scale adapted versions of standard cognitive tests were administered via a touchscreen interface. The following five tests were performed in the assessment centre: prospective memory (ID: 20018), pairs matching (number of incorrect matches: 399), fluid intelligence (20016), digit recall test (4282), and reaction time (20023). Additional cognitive tests were introduced during subsequent assessment centre visits, adding trial making test A (duration numeric: 6348, errors numeric: 6349) and B (duration alphanumeric: 6350, errors alphanumeric: 6351), symbol digit substitution (23324) and puzzles correctly solved (6373) to the test battery. In 2014, participants were also invited to take part in online cognitive tests, involving re-assessments of most assessment centre cognitive measures, including fluid intelligence (20191), trail making test A (duration numeric: 20156, errors numeric: 20247) and B (duration alphanumeric: 20157, errors alphanumeric: 20248), symbol digit substitution (20159), pairs matching (20132), digit recall test (20240) and puzzles correctly solved (20760). A detailed description of the development and application of the cognitive measures can be found elsewhere[1]. Since cognitive tasks were added and removed at different assessment occasions (c.f., **Supplementary Figure 15**), the number of participants with complete longitudinal (i.e., two waves) cognitive data varies across tests. To maximize the sample size for longitudinal analyses, we combined data obtained from the online follow-up and assessment centre visit (e.g., pairs matching test score assessed during the assessment centre visit at baseline and online at follow-up). For fluid intelligence, we recomputed the summary score obtained from the online version of the test, as this version includes an additional question that is not part of the fluid intelligence test administered during the assessment centre visit. Question 14 (alphanumeric substitution) from the online test was therefore not included in the summary score, such that the maximum test score was 13 for the two tests. Similarly, the trail making test scores obtained during the assessment centre visit (ID: 6349 and 6351) contained zero values, while the online version (ID: 20247 and 20248) recorded scores only for participants who made at least one error in that test. To make the two versions comparable, we therefore excluded individuals with zeros in the assessment centre version of that test. A number of measures (reaction time, trial making test A and B, pairs matching) were recoded so that higher values indicate better cognitive function.

For physical measures, we included only health variables that are subject to age-related decline, namely phenotypes that, on average, decline as people get older (e.g., grip strength). Excluded were phenotypes that

tend to vary over time but that do not necessarily show decline over time (e.g., pulse rate, body mass index). For indexes of physical functioning, we included forced expiratory volume (FEV, 3063), hand grip strength (taking the average of both the right (47) and left (46) hand), maximum workload during fitness test (6032), heel bone mineral density (4124), hearing test performance (average of the right, 20021, and left, 20019), and height (50). As seen in **Supplementary Figure 15**, all measures used for physical function in this study were obtained during the assessment centre visits, although the samples sizes with available longitudinal data varied.

In total, we included 11 cognitive and 6 physical measures. For a number of measures, including fluid intelligence, symbol digit substitution test, number of puzzles correctly solved and grip strength, we removed individuals with a score of zero. A score of zero in the aforementioned continuous cognitive and physical traits values may indicate measurement problems (e.g., inability to understand the instructions or handling the computer program for cognitive measures, or calibration errors leading to lower than expected values, as documented for grip strength) and were therefore discarded. To minimize the impact of possible outlier values, we also excluded baseline and/or follow-up observations with large deviations ($>$ 10 standard deviations) from the baseline mean.

When processing the longitudinal data, we re-structured the multiple-wave data for UKBB participants such that the first available data point per individual for a particular phenotype was set as the baseline measure for a particular phenotype, irrespective of whether this was assessed at the first assessment centre visit or at a later time point (e.g., for measures only introduced at later assessments). For individuals with more than two waves of assessments, we used data from the most recent assessment as the follow-up point, to maximize the follow-up duration and facilitate the detection of aging-varying genetic effects.

Evaluation of two-wave models of change to study longitudinal genetic effects

One important challenge when assessing age-varying genetic effects lies in how change over time is defined. For example, interactions can be scale dependent, where an interaction may be present on one scale (e.g., absolute change) but not on another (e.g., relative change)[2]. Additionally, adjusting for baseline differences using residual change (that is, baseline-adjusted) scores has been shown to introduce bias in observational studies when the exposure of interest is associated with baseline levels of the outcome variable[3]. In light of these considerations, we hypothesize the following with respect to risk of bias associated with different definitions of change:

1) Change adjusted for baseline values can correctly identify genetic effects on change in situations where the genetic variant is unrelated to the baseline phenotype
2) Change unadjusted for baseline values can correctly identify genetic effects on change even if the genetic variant is associated with baseline phenotype
3) Differences in findings obtained from absolute and relative change can arise due to scale dependencies

We performed a series of simulation analyses to assess risk of bias when using the three change definitions described above, including absolute (Δ_{DIFF}), conditional (Δ_{RES}) and relative change (Δ_{LOG}). We simulated data according to the structural causal model shown in **Figure 2** (main manuscript). As illustrated, the longitudinal (i.e., age-dependent) effects were conceptualized as gene $\times$ environment interactions, where the genetic effect differs across age-varying environments. The observed phenotype P^* at time point t (0 = baseline, 1 = follow-up) was modelled as follows:

$$P_t^* = P_t + \varepsilon_t = \lambda + \alpha \cdot G_0 + \beta \cdot E_t + \gamma \cdot G_E \cdot E_t + \varepsilon_t \tag{1}$$

where P_t is the phenotype free of measurement error, ε_t the measurement error in the phenotype, λ the intercept, α is the time-invariant (cross-sectional) genetic effect, G_0 the time-invariant (cross-sectional) genetics, β the environmental effect, E the environment, γ the gene $\times$ environment effect, G_E the time-varying (longitudinal) genetics.

Another realistic model underlying the observed data is a multiplicative one, which was modelled as:

$$P_t^* = \exp(P_t + \varepsilon_t) = \exp(\lambda + \alpha \cdot G_0 + \beta \cdot E_t + \gamma \cdot G_E \cdot E_t + \varepsilon_t) \tag{2}$$

We simulated the data from both models with the following parameters: The intercept (λ) was set to mimic a

continuous, non-negative, phenotype. We set $\lambda = 170$, indicative of measured height in cm as simulated from model (1). The genetic (G_0, G_E) and baseline environmental (E_0) variables were generated as Gaussian normal variables with zero mean and unit variance, such that $G_0 \sim \mathcal{N}(0, 1)$ and $G_E \sim \mathcal{N}(0, 1)$ are uncorrelated and $E_0 \sim \mathcal{N}(0, 1)$. Change in the environment ($\Delta_E = E_1 - E_0 \neq 0$) was modelled as a shift in the mean from baseline to follow-up plus a random change with unit variance [i.e. $\Delta_E \sim \mathcal{N}(0.1, 1)$]. We varied two parameters in the simulations, including the cross-sectional (baseline) genetic effect (α) and the degree of measurement error per phenotype at time point t (ε_t). Measurement error was simulated as $\varepsilon_t \sim \mathcal{N}(0, \sqrt{(1-\rho) \times \sigma_t^2})$, where ρ indexes the measurement reliability and σ_t^2 is the variance of the error-free phenotype at time point t [$\sigma_t^2 = var(P_t)$]. The maximum ε_t (i.e., when $\rho = 0$) is therefore equal to the standard deviation of P_t.

To evaluate the performance of the three definitions of change ($\Delta_{DIFF}, \Delta_{RES}, \Delta_{LOG}$), we applied them to data generated based on model (1) and (2). Our first aim was to assess the different change definitions for their ability to distinguish cross-sectional (α) from longitudinal genetic effects (γ). To that end, we focused on the observed association of G_0 on change ($\Delta^* = \pi \cdot G_0 + \varepsilon$) to quantify risk of bias. As the true causal effect of G_0 on change is $\pi = 0$ (c.f., structural causal model **Figure 2** in the main manuscript, with cross-sectional genetics of P_1^* and P_0^* cancelling each other out), a non-zero observed effect ($\hat{\pi} \neq 0$) reflects a spurious association between cross-sectional genetics and change. The following linear regression models were tested in simulations to obtain $\hat{\pi}$ on change, including (1) difference scores ($\Delta_{DIFF}^* = P_1^* - P_0^* = \pi \cdot G_0 + \varepsilon$), (2) residual change scores ($\Delta_{RES}^* = P_1^* - P_0^* = P_0^* + \pi \cdot G_0 + \varepsilon$) and (3) log-difference scores where true change was simulated as exponential ($\Delta_{LOG}^* = ln(P_1^*) - ln(P_0^*) = \pi \cdot G_0 + \varepsilon$). Within this setting, we also evaluated the impact of model misspecification (Δ_{DIFF} is designed to be fitted to model (1) and Δ_{LOG} is designed to be optimal for model (2)).

For each scenario, we evaluated risk of false positives, based on the proportion of simulation repetitions where the null hypothesis is rejected (using a 5% level of significance) when testing the baseline genetic effects on change ($\Delta^* \sim \pi \cdot G_0$). Power was quantified by obtaining the true-positive rate (at 5%) when testing for longitudinal genetic effects on change ($\Delta^* \sim \tau \cdot G_E$). In all scenarios, data was simulated for a sample of $N = 10,000$ and the cross-sectional and longitudinal genetic effects on change ($\hat{\pi}$ and $\hat{\tau}$, respectively) were obtained in $n_{sim} = 10,000$ repetitions. Parameters β and γ were set to $\beta = 1$ and $\gamma = 0.5$ in all simulations.

Sample representativeness and correction for selective participation

To assess the level of sample representativeness of the prospective UKBB sample, we (1) compared UKBB characteristics to those of a representative reference sample and (2) assessed phenotypic and genotypic factors differentiating dropout from follow-up participants within the UKBB. With respect to (1), we used a previously curated external dataset (Health Survey England, HSE[4]) containing 14 variables related to health, lifestyles, education and basic demographics information that were harmonized with the UKBB data[5]. Using that data, we performed univariate logistic regression predicting UKBB baseline participation (HSE=0; UKBB baseline sample = 1) and follow-up participation (HSE=0; UKBB follow-up sample = 1) to estimate the contribution of each of the 14 harmonized variables to levels of non-representativeness. To identify features differentiating dropout from follow-up participants (2), we assessed if participation status (0=dropout; 1=included) was predicted by UKBB baseline characteristics, including the cognitive and physical aging phenotypes used in this work. Further, we explored if differences between the two groups (dropout versus included) were present at the genetic level, by performing genome-wide scans on the baseline cognitive and physical measures in the two groups separately. The association tests were performed in REGENIE (v3.2.6) (details in the main manuscript) and differences in genomic findings were quantified in terms of differences in SNP-heritability and genetic correlations using LD score regression[6].

To explore risk of bias due to selective participation, we employed inverse probability weighting (IPW) to create a pseudo-sample of the UKBB with higher levels of representativeness. As sampling weights, we used previously generated UKBB participation probabilities designed to adjust for selective baseline participation[5], and we generated a second set of probability weights to adjust for selective follow-up participation. The follow-up probability weights were obtained from a LASSO regression model (as described here[5]) predicting UKBB follow-up participation (HSE=0; UKBB follow-up sample = 1), based on the same 14 harmonized variables used to generate the baseline participation weights. The two sets of probability weights were then included in weighted least squares regression (WLS) when testing phenotype associations (using WLS as implemented in the R Package *survey*[7]) and genotype association (using WLS as implemented in LDAK version 5.2[5,8]).

Supplementary Discussion

Slopes obtained from three-wave data

To evaluate whether adding an additional time point improves the measurement of change, we derived slopes of change for one phenotype (fluid intelligence) with at least three non-missing observations (N = 69,801). First, we applied a linear mixed model (LMM) with random intercepts and slopes to estimate individual slopes of change. However, the LMM resulted in singular fits, suggesting that the model structure was too complex for the data. We therefore switched to ordinary least squares (OLS) regression. For each individual with three-wave data, we regressed the phenotype (Y_t) at time point t on the years elapsed since baseline ($t = 0$) using the model $Y_t \sim \text{years}_t$. The individual regression coefficients were then taken as the three-wave slopes of change.

We then compared the three-wave slopes (Δ_{W3}) to two-wave slopes of change ($\Delta_{W2} = \frac{\Delta_{P_1 - P_0}}{FU}$), which were derived based on the difference between the phenotype assessed at baseline (P_0) and the most recent follow-up assessment (P_1), divided by the follow-up time (FU). Comparing the two slopes of change (Δ_{W2} and Δ_{W3}) revealed a high correlation between the two (r = 0.96), and the addition of one more time point increased the between-subject variance from 41.4% to 48.2%, indicating improved precision in Δ_{W3}. Specifically, the total variance of the phenotype is composed of $Var(P) = \sigma_I^2 + \sigma_V^2 + \sigma^2$, where σ_I^2 corresponds to the time-invariant, between subject variance (e.g., genetic), σ_V^2 to the time-variant (within-subject) change and σ^2 to the measurement error. Incorporating additional time points would reduce measurement error (σ^2), thereby increasing the proportion of the variance explained by between-subject differences ($\frac{\sigma_I^2}{\sigma_I^2 + \sigma_V^2 + \sigma^2}$).

In summary, the additional time point modestly improved the precision of the slope of change ($\sim$ 6.8%), which has implications for the estimation of variance components (e.g., h^2 of change) and statistical power to detect age-varying genetic effects. However, the larger sample size available from two-wave data for fluid intelligence (159,762), along with greater sample representativeness, likely outweighs the modest precision gains achieved by the incorporation of the additional time point.

Sample representativeness and correction for selective participation

We performed a number of sensitivity checks to characterize features of follow-up participation and risk of bias due to selective attrition. First, comparing the baseline and follow-up UKBB sample to a representative reference sample (**Supplementary Figure 19**), we observed that UKBB participation was largely driven

by social, health and lifestyle factors. While UKBB baseline participation (subscript B) and participation in follow-up research (subscript FU) were predicted by similar factors, larger effect estimates were observed for factors predicting follow-up participation (e.g., $\beta_B = 0.09$ versus $\beta_{FU} = 0.26$ for education), explaining in total almost twice as much of the variance in the respective participation behaviour ($R^2_B = 12\%$ versus $R^2_{FU} = 23\%$). Second, as illustrated in **Supplementary Figure 19**, baseline levels of many phenotypes used as a proxy for physical and cognitive aging predicted participation status (0=dropout; 1=follow-up participation): $\beta_{\text{fluid intelligence}} = 0.26$, $\beta_{\text{working memory}} = 0.14$, $\beta_{\text{FEV}} = 0.11$ and $\beta_{\text{fitness}} = 0.07$. Subtler differences were observed in the effects of many genetic markers on cognitive and physical measures when the associations were performed separately in the dropout and the follow-up sample (**Supplementary Figure 20**). For example, for most measures, the genetic correlation between effects obtained in the two sub-samples remained close to one (ranging between $r_g = 0.98$ and $r_g = 1.03$).

To assess the impact of the observed non-random follow-up participation, we applied Inverse Probability Weighting (IPW). **Supplementary Figure 21** shows the distribution of the baseline and follow-up sampling weights (w_B and w_{FU}, respectively) used in IPW. While we observed a large correlation between the two weights ($r = 0.77$), the follow-up weights showed greater variability than the baseline weights [$\sigma^2(w_{FU}) = 5.48$ versus $\sigma^2(w_B) = 1.76$, respectively], resulting in a greater loss in effective sample size (**Supplementary Figure 21**). Applying IPW, we found that, at the phenotypic level (**Supplementary Figure 1**), bias due to selective participation appeared most visible for the cross-sectional age effects on measures of cognitive and physical function. As illustrated in **Supplementary Figure 1**, selective participation resulted in attenuated age effects, where attenuation bias aggravated with increasing rates of loss to follow-up and non-representativeness. At the genotypic level, we performed weighted genome-wide tests for all variants significantly ($P < 5 \times 10^{-8}$) associated with cognitive and/or physical outcomes (described in the next section) and compared the variant effects to those obtained from standard GWA. **Supplementary Figure 11** shows that bias was present in both directions, leading to over-estimation (e.g., *APOE*-effects on cognitive-Δ) and under-estimation (e.g., *DUSP6*-effects on physical-Δ) of variant effects. Across all tested variants, we did not, however, find evidence of altered direction of effects.

Evaluation of two-wave models of change to study longitudinal genetic effects

We performed a series of simulation analyses to assess the suitability of two-wave models of change for the estimation of longitudinal genetic effects, including difference scores (Δ_{DIFF}, indexing absolute change), log-difference scores (Δ_{LOG}, indexing relative change) and residual change scores (Δ_{RES}, i.e., baseline-adjusted

absolute change) (**Supplementary Figure 6**). In brief, we found that Δ_{RES} induced inferential errors and false-positive associations in most simulated scenarios, by falsely associating cross-sectional (time-invariant) genetic effects with change. Unbiased effects were only identified in scenarios where the cross-sectional (time-invariant) genetic effect on the phenotype was equal to zero ($\alpha = 0$, Panel A1 in **Supplementary Figure 6**) and/or there was no measurement error in the phenotype ($\rho = 1$, Panel B1). Further, the degree of bias varied as a function of the reliability of the phenotype (Panel A) and the cross-sectional genetic effect on the phenotype (Panel B). As illustrated visually in **Figure 1** in the main manuscript, bias via baseline-adjustment can result from collider-stratification, i.e., the conditioning of P_0^* as a common consequence of the cross-sectional genetics and the measurement error ($G_0 \rightarrow P_0^* \leftarrow \varepsilon_0$). In contrast, Δ_{DIFF} and Δ_{LOG} robustly separated cross-sectional from longitudinal genetic effects in the simulated scenarios, irrespective of the contribution of the cross-sectional genetic effect or the degree of measurement error in the phenotype.

Panel C/D (**Supplementary Figure 6**) illustrate how model misspecification can impact the interpretation of the regression parameters, for example when the assumed function of change does not align with its underlying function. We found that using absolute change as an outcome (Δ_{DIFF}) to data simulated under the exponential model inflated the false positive rate (up to 7%, highlighted in dark blue in Panel C), resulting in a non-zero cross-sectional genetic effect on change. Conversely, using relative change as outcome (Δ_{LOG}) to data simulated from the linear change model maintained a well-controlled Type I error rate at 5% (highlighted in dark orange), albeit reduced power to detect longitudinal genetic effects. Together, the results suggest that Δ_{LOG} is more robust to the type of model misspecification considered here. Finally, the use of Δ_{RES}-scores resulted in biased estimates irrespective of whether a correctly or incorrectly specified model was fitted to the data (with false positive rates of 22% and 8%, respectively), reiterating that bias from baseline-adjustment is likely to occur in many real-world scenarios.

To further illustrate the impact of model choice on the interpretation of longitudinal effects, we obtained phenotypic associations between a number of UKBB baseline characteristics with global cognitive and physical decline (**Supplementary Figure 7**). While results from absolute and relative change used as outcomes converged for most exposure-outcome estimates (e.g., male sex linking to increased cognitive decline, with $r_{\Delta_{DIFF}} = 0.04$ and $r_{\Delta_{LOG}} = 0.03$), noticeable scale-dependencies were observed for the association of sex with physical decline ($r_{\Delta_{DIFF}} = 0.05$ and $r_{\Delta_{LOG}} = -0.1$). This pattern of scale-inconsistencies therefore points to the presence of non-linear change, where individuals with more extreme baseline values show steeper absolute (but not relative) change compared to individuals closer to the average.

Of note, inconsistencies arising from varying definitions of change have also been discussed in clinical trials employing pre-post designs[9]. In trials with balanced baseline values in the outcome variable, both absolute change and baseline-adjusted change (e.g., ANCOVA) provide unbiased estimates of the treatment effect. In such cases, ANCOVA is often preferred for its potential to increase statistical power[10,11]. However, the assumption of baseline balance is rarely met in absence of randomization, and may still be violated under randomization due to chance imbalances[12]. In those situations, baseline adjustment will provide biased results and has therefore been discouraged[10,11,13]. The same conclusion has been drawn with respect to the use of baseline-adjusted change scores in trials on pharmacogenetic effects, where the induced bias is proportional to the baseline genetic effect (c.f., Supplementary Note 1 in Ref[14]). Therefore, interpreting results from baseline-adjusted models should be contingent on verifying baseline balance in clinical trials.

In summary, our findings highlight that baseline-adjusted change — despite its widespread use — tend to introduce bias and false-positive associations when estimating age-varying genetic effects. Conversely, models capturing absolute and relative change are more robust in distinguishing cross-sectional from longitudinal genetic effects under various realistic conditions. However, as scale-dependencies cannot be ruled out, using both models together can help with the interpretation of findings. In subsequent analyses, we therefore prioritized definitions of absolute and relative change when scrutinizing longitudinal genetic effects.

Reserach in context

A main goal of life course epidemiology is to elucidate the structure of the aging process and to identify factors that promote healthy aging. One central focus lies on understanding individual differences in age-related decline across different domains of functioning, as marked inter-individual variation characterizes the pace at which individuals age. In this context, genetic factors are increasingly studied, given their promise to directly (e.g., via molecular/pharmacological targets) or indirectly (e.g., via environmental targets) provide insights into aetiology and prevention.

Conceptually, genetic effects on age-related decline represent time-varying genetic effects or gene-environment interactions, where the genetic effects differ across changing environments as individuals age[15]. Different methodological approaches have been employed to identify age-varying genetic effects. For example, large-scale cross-sectional studies have tested for gene-by-age interactions[16–20]. These studies leverage the full age spectrum available at a single point in time (e.g., ages 40 to 69 in the UK Biobank) when testing for gene-by-age interactions. However, a key limitation of cross-sectional gene-by-age designs lies in their inability to rule

out potential cohort effects[15], as stratifying individuals by age groups inherently stratifies groups by their year of birth. Given these and other limitations (e.g., possible non-linear age effects) associated with cross-sectional gene-by-age analyses, longitudinal GWA are considered a more robust approach for investigating time-varying genetic effects. In absence of large-scale longitudinal biobank data, early research has primarily focused on candidate genes[21,22] or genome-wide effects on longitudinal change in smaller genotyped samples (<2000[23–27]). More recently, with the release of large samples with repeat assessments (i.e., >100,000 individuals), genome-wide studies on longitudinal changes have become more feasible, enabling the examination of genetic influences on changes in routinely collected healthcare data (e.g., BMI[28] or biomarker changes[29]) and health outcomes measured through repeated research assessments.

Studying age-varying genetic effects is important for a number of reasons. First, such work allows to assess if the genetic architecture underlying lifetime levels of function is similar to that characterizing age-related change. In other words, while tests on change quantify the impact of age on genome-wide associations, cross-sectional genome-wide analyses identify marginal genetic effects that are assumed to be constant over time (c.f., study framework, **Figure 1** in the main manuscript). Second, genetically informed designs allow to further probe existing theoretical models of aging. For example, one key interest concerns the question as to whether 'age is kinder to the initially more able'[30–32], as innate and/or early life resources may slow down age-related decline. Similar prediction are made based on theories of 'cognitive reserve'[33], 'brain reserve'[34], 'health capital'[35] or 'differential preservation'[36], broadly hypothesizing that individuals with higher levels of reserve are more protected from neurobiological decay throughout the aging process. Evidence on this topic has, however, largely been mixed: While some studies suggest that health reserves may protect against age-related decline, including evidence linking educational attainment to slower cognitive decline[37–40], a larger body of research has failed to replicate these findings[41–49]. Our findings are consistent with the latter body of research, suggesting that high baseline function in a given trait (e.g., cognition) or related factors (e.g., educational attainment) may not buffer against age-related decline in that trait. In other words, while cognitive or physical reserves can delay the onset of functional impairment, they are unlikely to slow the overall rate of decline. Other lines of research have focused on common cause theories of aging, investigating whether age-related declines in physical and cognitive domains are driven by shared aetiological processes[50–53]. While small but significant associations between cognitive and physical decline have indeed been documented[50], findings remains largely inconsistent[43,50,53–56]. In our work, we observed some indications of shared risks (e.g., shorter parental lifespan linking to both increased cognitive and physical decline) when tested in Mendelian Randomization analyses. However, most implicated risk factors demonstrated independent effects across the

physical and cognitive dimensions of decline.

With respect to other health-related risk factors, previous observational research has mostly focused on cardiovascular risk factors[57,58], lipid traits[59], brain health[60], lifestyle factors (e.g., physical activity[61,62], alcohol use[63,64], smoking[65], diet[66]), mental health (e.g., depression[67,68], sleep[69,70]), early life factors (e.g., birth weight[71]) or social factors (loneliness[72], lower socioeconomic status[73]) as possible risks involved in cognitive or physical decline. While numerous of those factors also associated with decline in our phenotypic analyses, many of those did not survive stringent covariate adjustment in MR analysis, indicating possible confounding effects. Focusing on risk factors implicated by MR, our findings converge with experimental evidence reporting possible cognition-enhancing effects of higher vegetable intake[74] and increases in physical function following improvements in sleep and metabolic rate[75]. Other identified risk factors reflected markers of general vulnerabilities that are not directly amenable to intervention without additional knowledge on the specific pathways involved, such as the effect of Alzheimer's liability on cognitive decline or the effect of telomere length on physical decline. Our finding that longer paternal lifespan was predictive of both reduced physical decline as well as better lifetime physical function is in line with the view that genetic and environmental factors transmitted across generations can influence the offspring's ability to maintain health during the aging process[76–78].

The presented results should be interpreted in light of a number of limitations. First, age-related change was assessed using data from only two time points per individual, which reduces measurement precision compared to approaches utilizing more intensive longitudinal data. Curating intensive longitudinal data, for example via the use of hospital record data or the implementation of additional repeat assessments in the UKBB study protocol would therefore help to refine and enhance the definitions of change used in this work. Second, despite the large sample size, statistical power likely remains an issue. Genetic interaction effects are inherently small and harder to detect compared to marginal effects[79]. This limitation may also have hindered the identification of causal factors of decline in Mendelian Randomization (MR) analyses, potentially explaining why some factors were identified in phenotypic but not MR analyses. Larger studies are therefore warranted to evaluate whether these discrepancies are indicative of residual confounding in classical epidemiological research, or reflect insufficient statistical power. Third, while this study focused on commonly examined risk factors for age-related decline, further research is needed to elucidate the specific pathways through which the identified risks contribute to cognitive and physical decline over time. This is particularly important for factors that are not directly modifiable, such as sex, telomere length, parental lifespan, or genetic liability for Alzheimer's disease. Finally, the use of genetically informed designs restricts our analysis to risk factors that can be

instrumented with genetic variants. Important environmental and social correlates of decline that are not instrumentable, such as air pollution[80], stressful life events[81,82], or housing conditions[83], therefore require alternative causal inference methods when put to scrutiny in observational studies.

Supplementary Figures

Supplementary Figure 1. Age effects across measures of physical and cognitive function

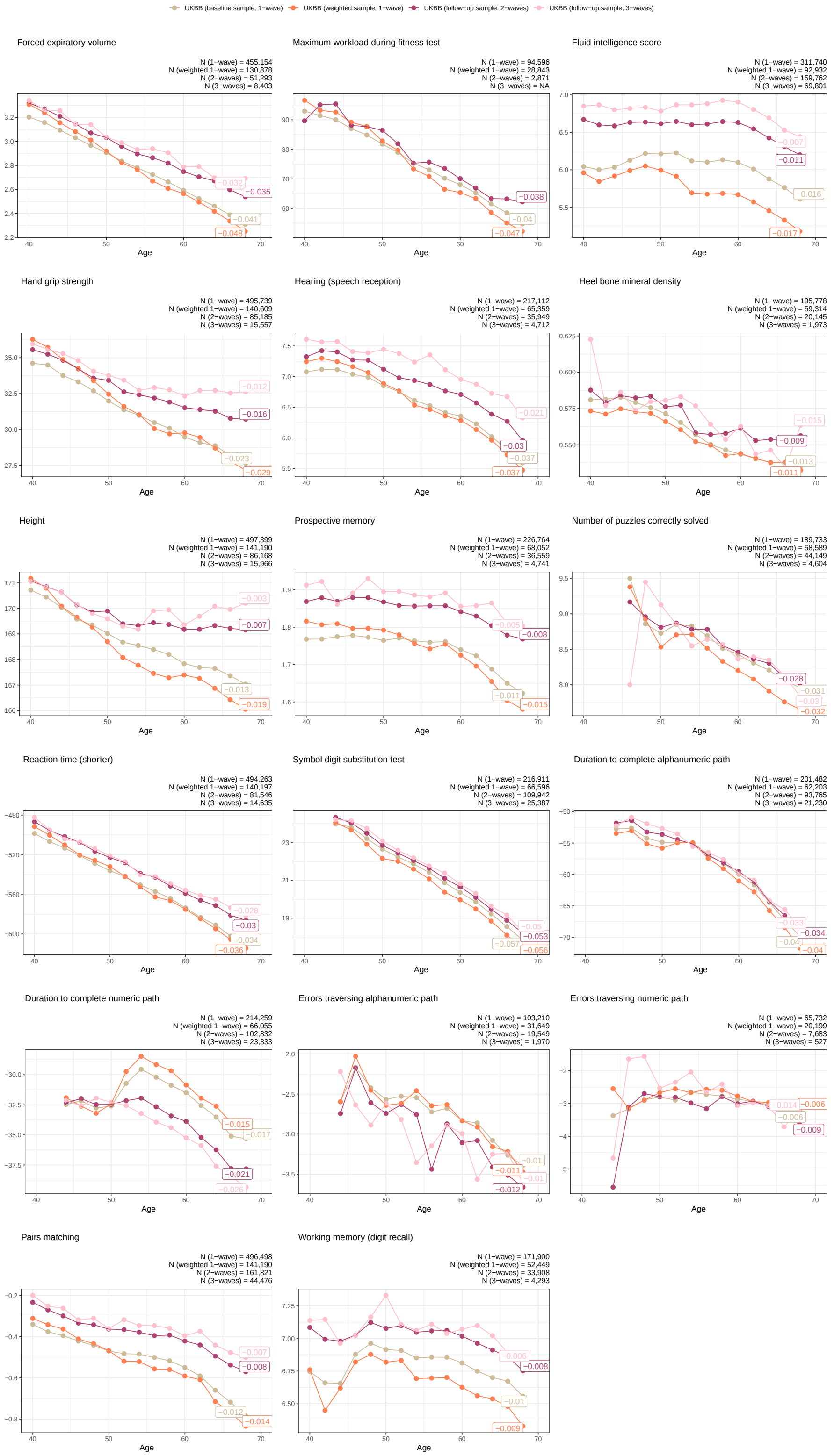

Supplementary Figure 1. Age effects across measures of physical and cognitive function. Each dot represents the mean phenotype score assessed at baseline (y-axis) per 2-year age bin (x-axis) across UKBB samples with varying levels of representativeness. Age effects (c.f., labels to the right) on cognitive and physical measures assessed at baseline were obtained from linear regression models, applied in a) the unweighted UKBB baseline sample ('baseline sample, 1—wave'), b) the inverse probability weighted UKBB baseline sample ('weighted 1—wave'), c) the UKBB follow-up sample with complete data in at least one follow-up assessment ('follow—up sample, 2—waves') and d) the UKBB follow-up sample with complete data in at least two follow-up assessments ('follow—up sample, 3—waves').

Supplementary Figure 2. Box and scatter plot of cognitive measures included in genome-wide scans

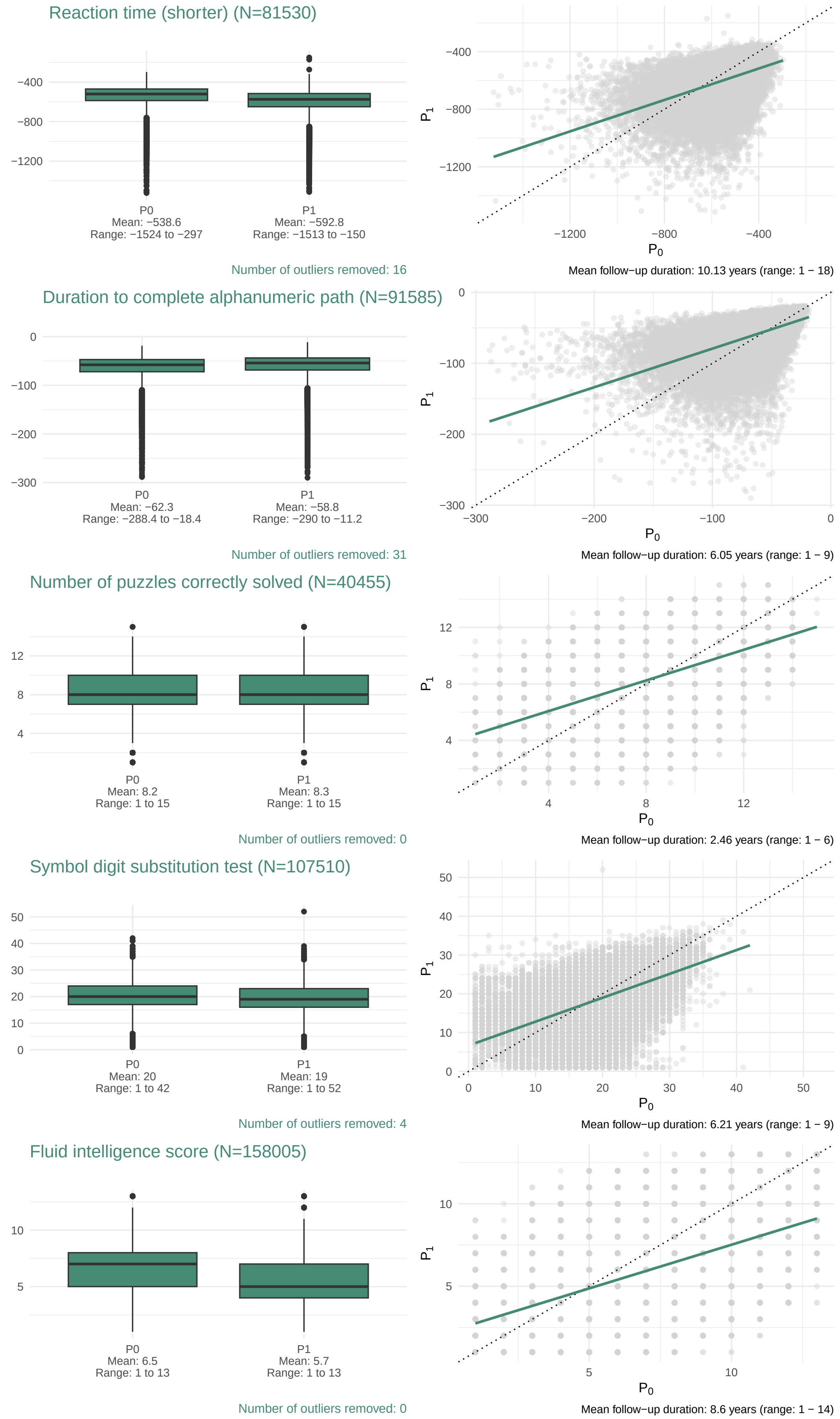

Supplementary Figure 2. Box and scatter plot of cognitive measures included in genome-wide scans. Box and scatter plots of cognitive measures assessed at baseline (P_0) and follow-up (P_1)

Supplementary Figure 3. Box and scatter plot of physical measures included in genome-wide scans

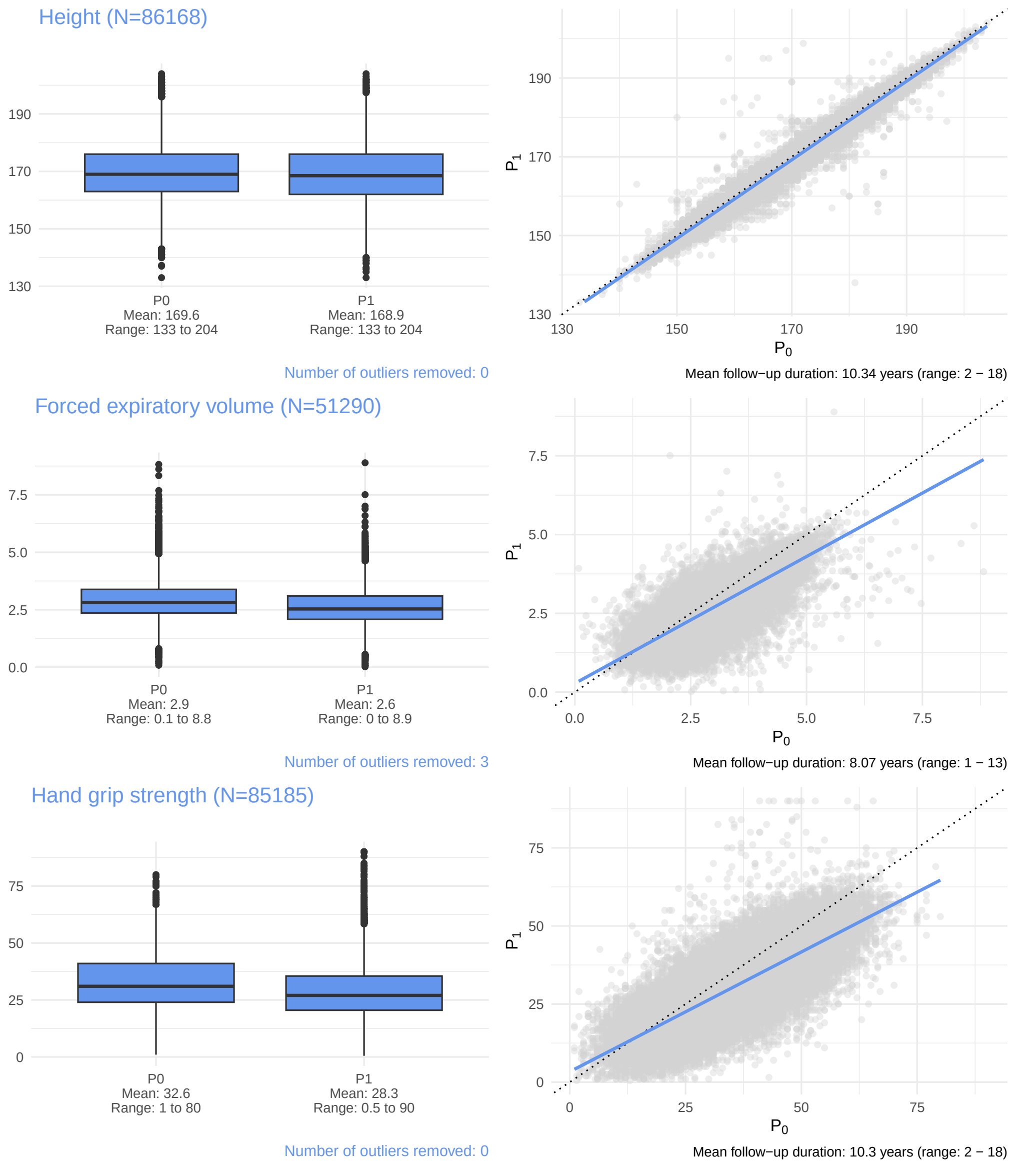

Supplementary Figure 3. Box and scatter plot of physical measures included in genome-wide scans. Box and scatter plots of physical measures assessed at baseline (P_0) and follow-up (P_1)

Supplementary Figure 4. Correlations between change scores indexing cognitive and physical decline

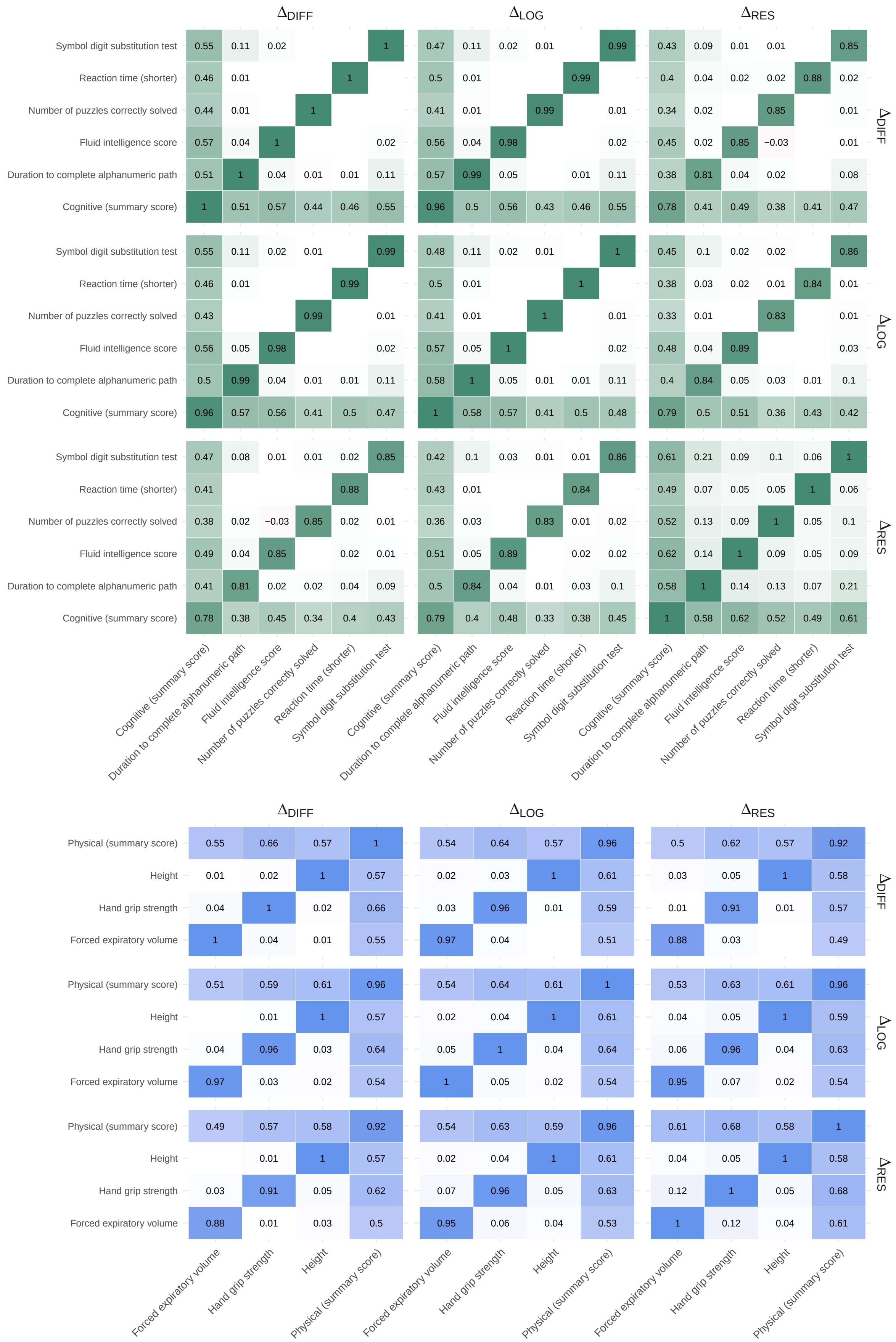

Supplementary Figure 4. Correlations between change scores indexing cognitive and physical decline. Correlation coefficients between indexes of decline, including absolute change (Δ_{DIFF}), conditional change (Δ_{RES}) and relative change (Δ_{LOG}). Only significant correlation coefficients per trait pair ($P < 0.05$) are shown.

Supplementary Figure 5. Correlations between baseline scores of cognitive and physical measures

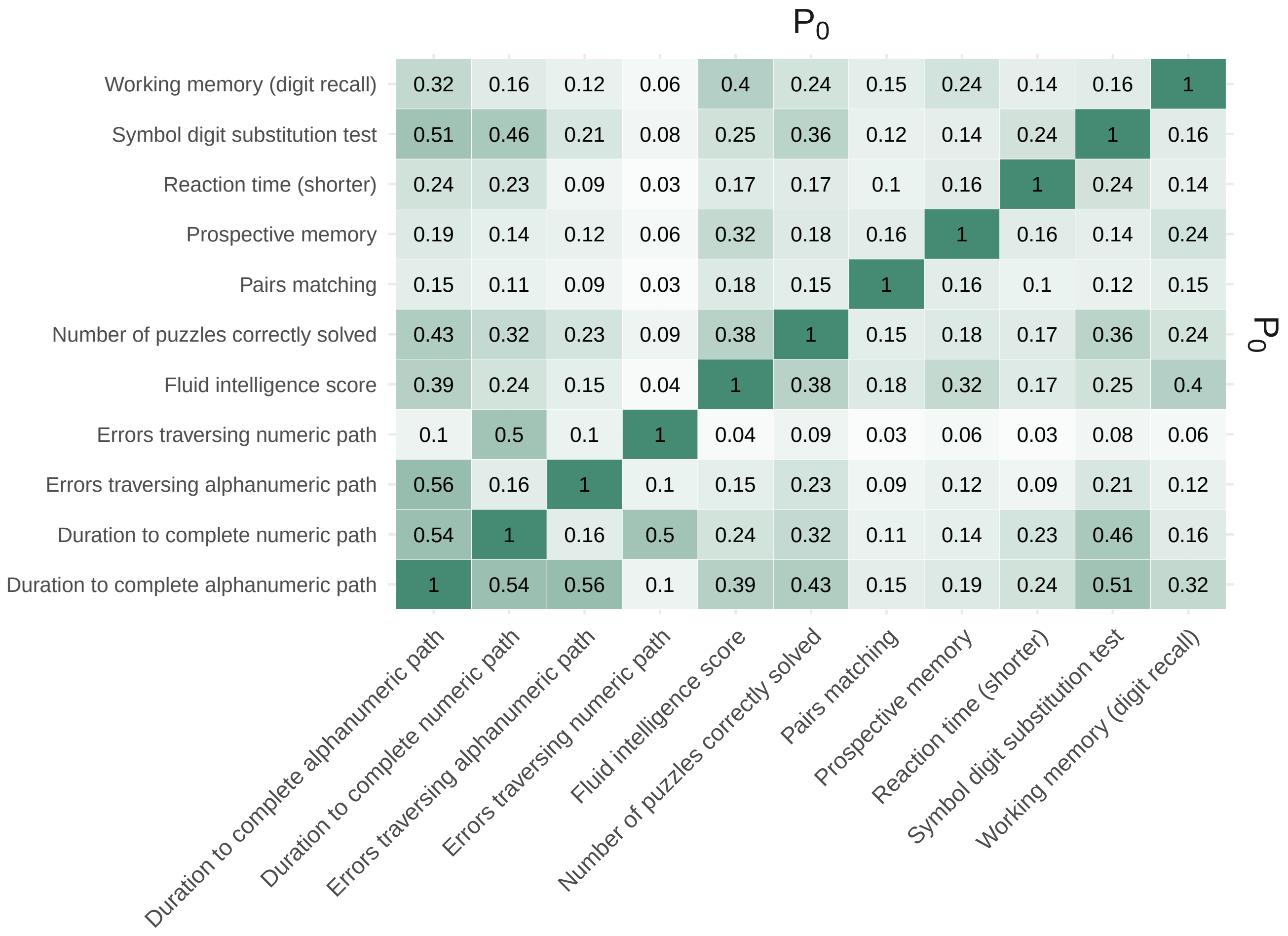

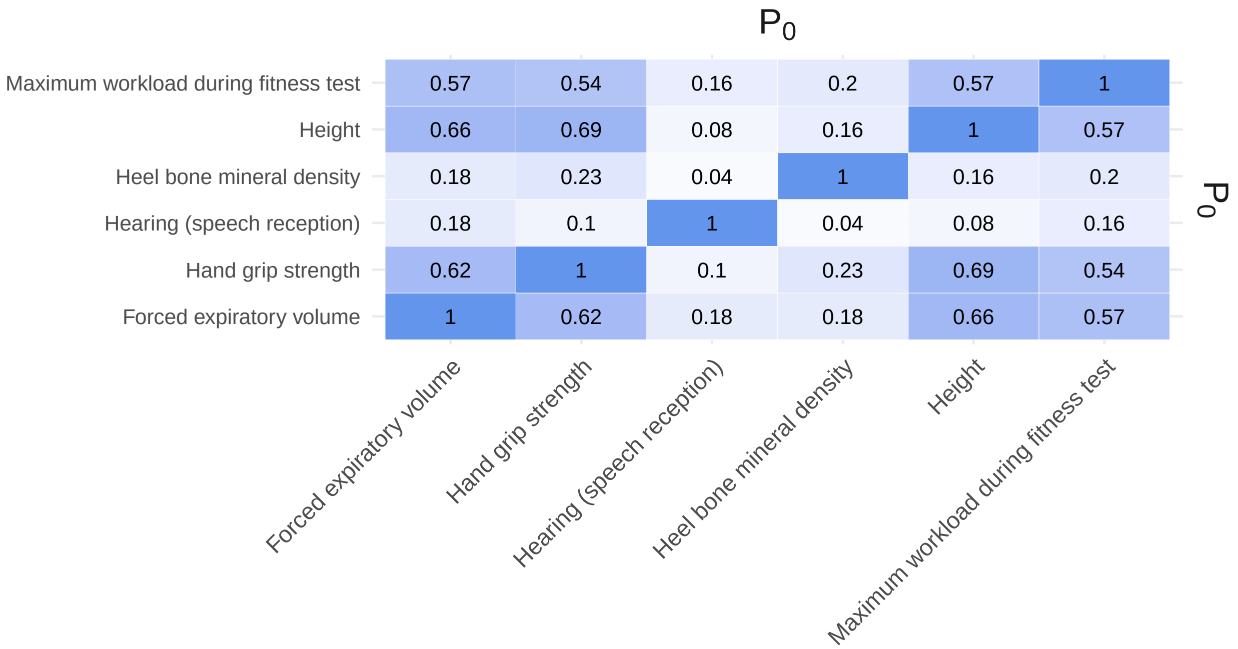

Supplementary Figure 5. Correlations between baseline scores of cognitive and physical measures. Correlation coefficients between cross-sectional physical and cognitive measures obtained at baseline. All measures were coded such that higher values index better physical/cognitive outcomes. Only significant correlation coefficients per trait pair ($P < 0.05$) are shown.

Supplementary Figure 6. Evaluation of two-wave models of change for longitudinal genetic effect estimation

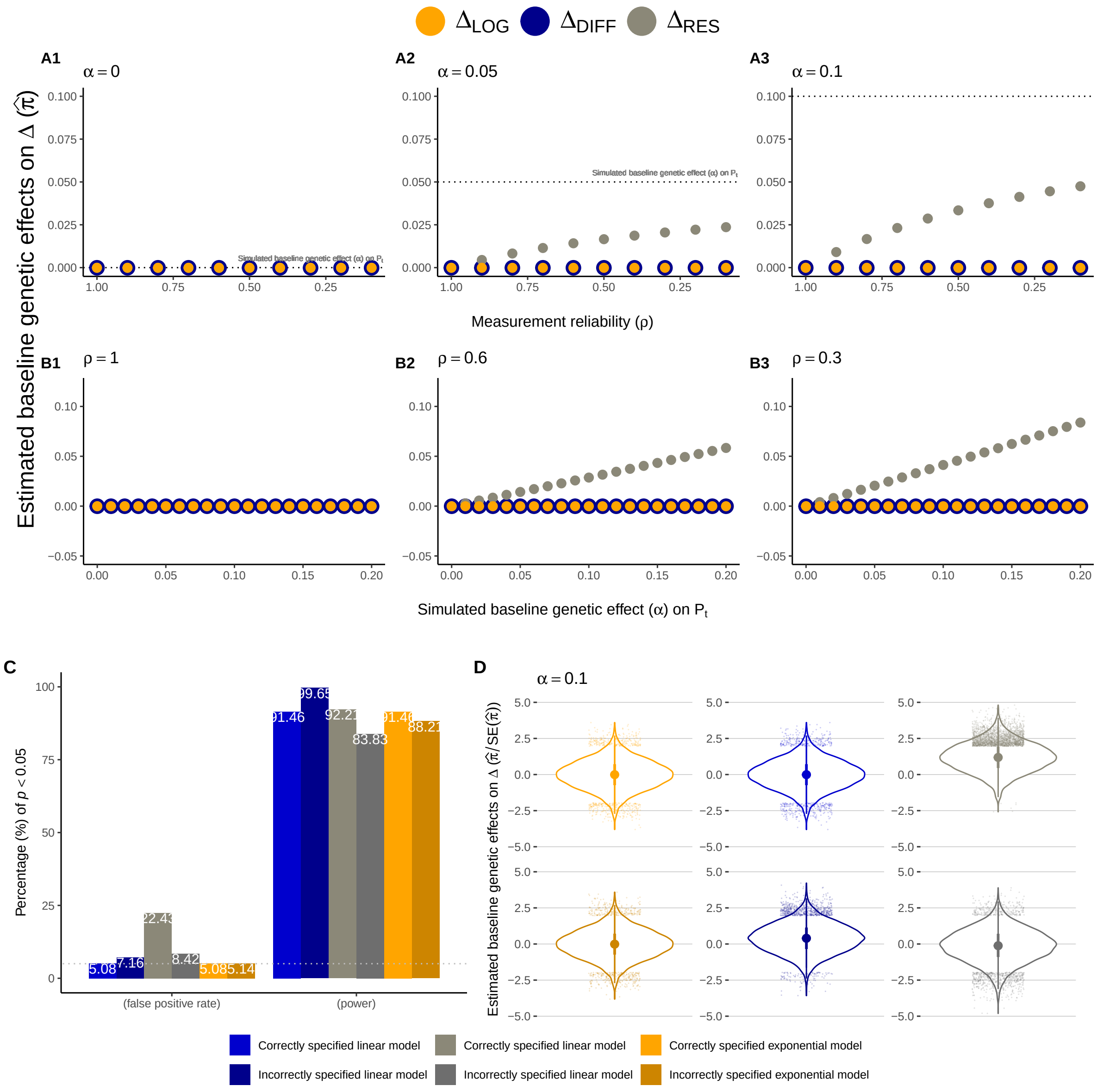

Supplementary Figure 6. Evaluation of two-wave models of change for longitudinal genetic effect estimation. Simulation results assessing risk of bias when using difference scores (Δ_{DIFF}), log-difference scores (Δ_{LOG}) and residual change scores (Δ_{RES}) for longitudinal genetic effect estimation. Data was simulated based on the model displayed in Figure 2 (main manuscript). **Panels A1-A3** and **Panels B1-B3** plot the observed baseline genetic effect on change ($\hat{\pi}$, obtained from $\Delta^* \sim \pi \cdot G_0$) across varying levels of measurement reliability (ρ, x-axis in Panel A1-A3) and baseline genetic effects on the phenotype (α, x-axis in Panel B1-B3). **Panel C** displays the rates of false positives (in %) when estimating the baseline genetic effect on change ($\Delta^* \sim \pi \cdot G_0$) and the true positive rate (power, in %) when estimating the longitudinal genetic effect ($\Delta^* \sim \tau \cdot G_0$) per model. The dashed line indicates the 5% significance level. The legend at the bottom lists the six models fitted to obtain $\hat{\pi}$ and the corresponding P-values. **Panel D** plots the z-scores ($\frac{\hat{\pi}}{SE(\hat{\pi})}$) obtained from the six models, highlighting all significant ($P < 0.05$) coefficients.

Supplementary Figure 7. Phenotypic predictors of change in the UK Biobank

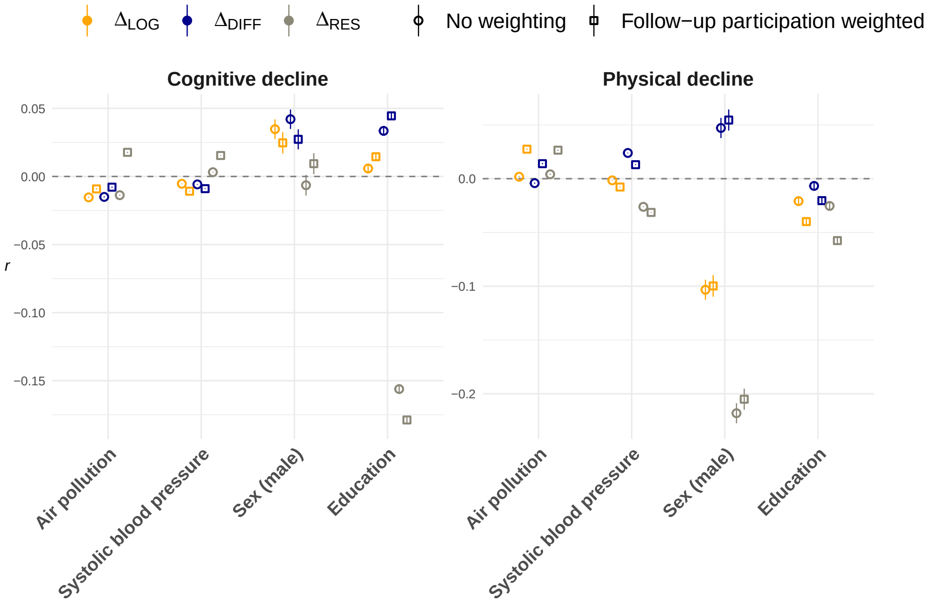

Supplementary Figure 7. Phenotypic predictors of change in the UK Biobank. The figure plots phenotypic associations between selected UKBB baseline characteristics and indexes of global cognitive and physical decline as derived from the the three definitions of change. The pearson correlation coefficients (r) were estimated in the unweighted UKBB follow-up sample and the inverse probability weighted sample.

Supplementary Figure 8. Longitudinal and cross-sectional genetic effects on cognitive and physical function

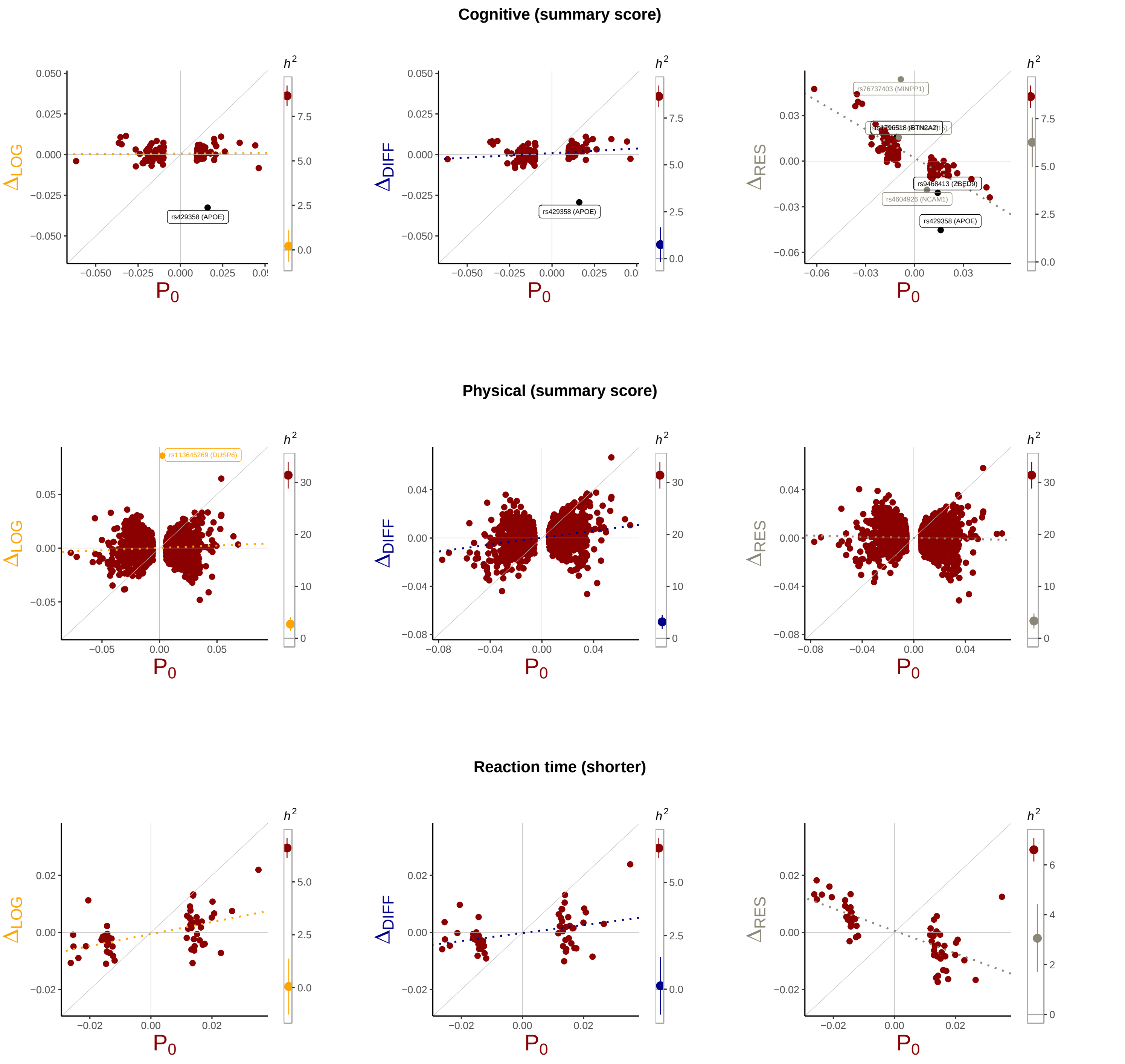

Duration to complete alphanumeric path

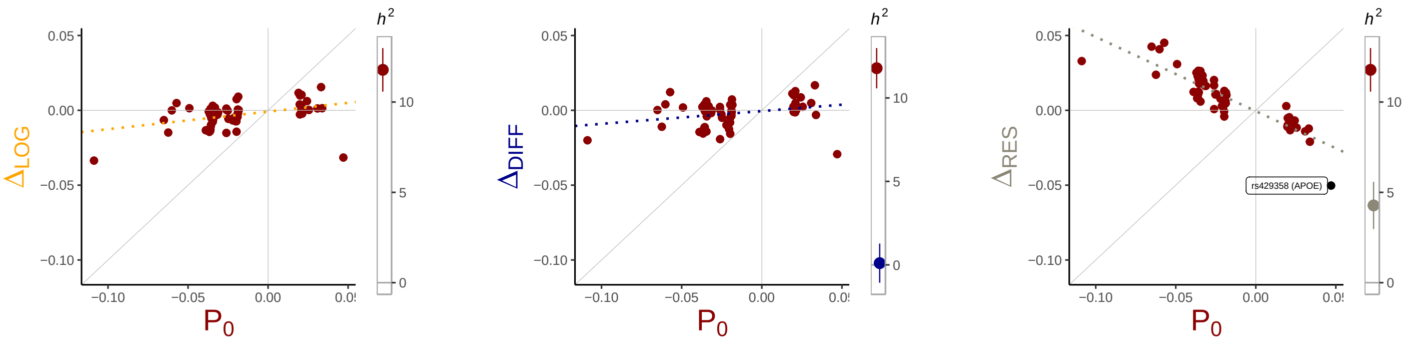

Number of puzzles correctly solved

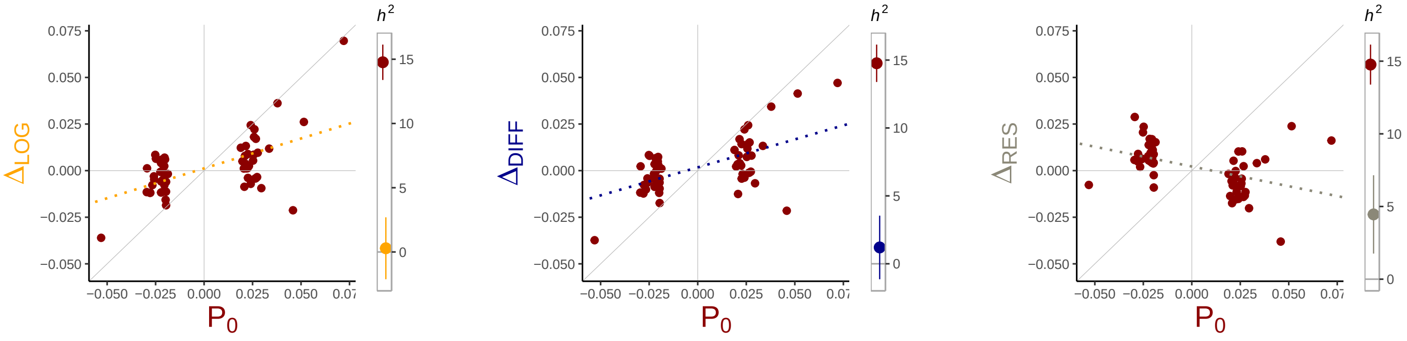

Symbol digit substitution test

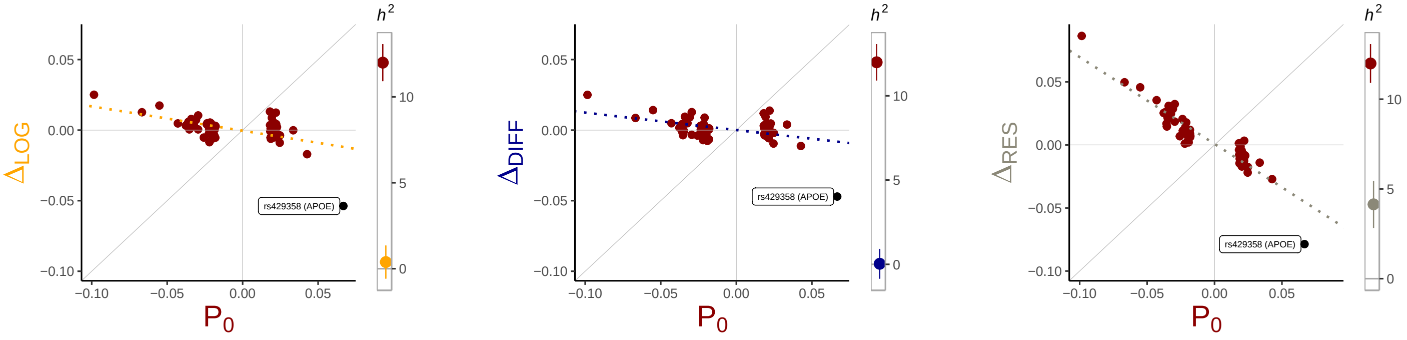

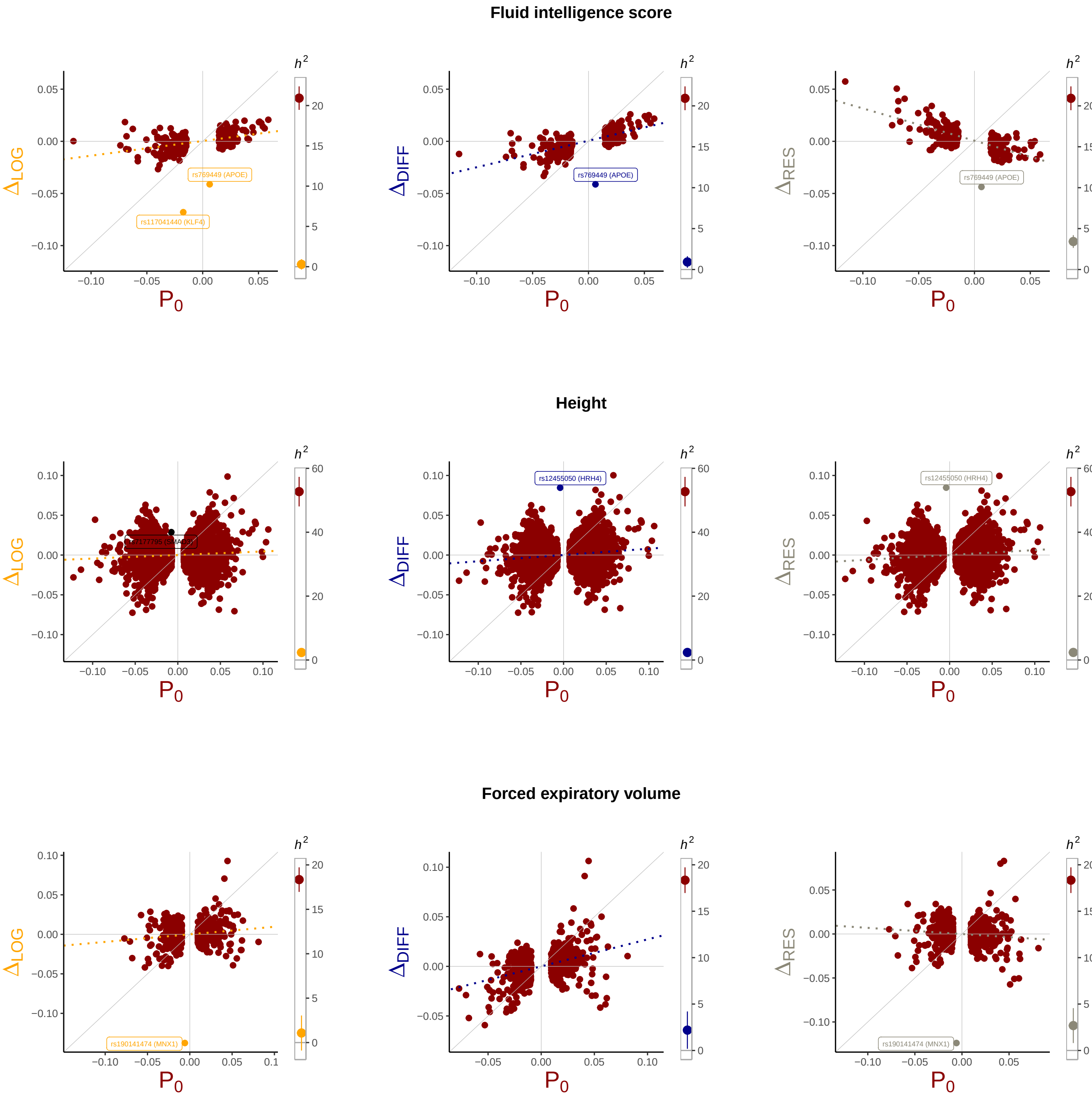

Fluid intelligence score
Height
Forced expiratory volume
ΔLOG
ΔDIFF
ΔRES
P0
h2
rs769449 (APOE)
rs117041440 (KLF4)
rs12455050 (HRH4)
rs190141474 (MNX1)

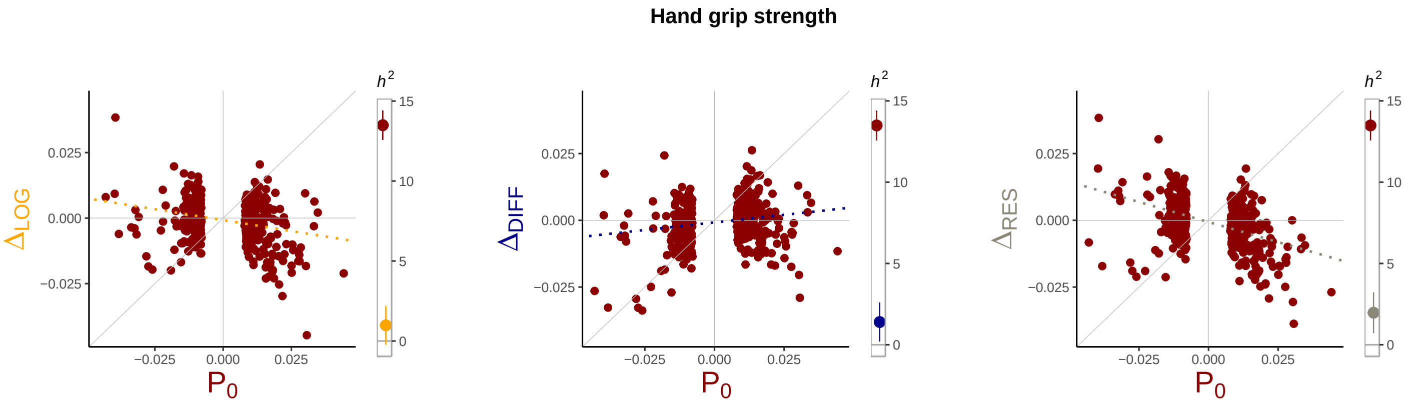

Supplementary Figure 8. Longitudinal and cross-sectional genetic effects on cognitive and physical function. Effects estimates of variants reaching genome-wide significance in association tests on either cross-sectional physical/cognitive functioning (x-axis) or cognitive/physical decline (y-axis). The colour scheme highlights variants associated with either cross-sectional (P_0 in red), longitudinal (Δ_{LOG} in orange, Δ_{DIFF} in blue, Δ_{RES} in grey) or both (in black) outcomes. The dashed slope (line of best fit) represents the association between the cross-sectional and longitudinal SNP effects. The SNP-heritability estimates (h^2, with 95% confidence intervals) obtained from longitudinal and cross-sectional genome-wide analyses are shown to the right of each panel.

Supplementary Figure 9. Genotype-phenotype associations of variants associated with cognitive or physical decline

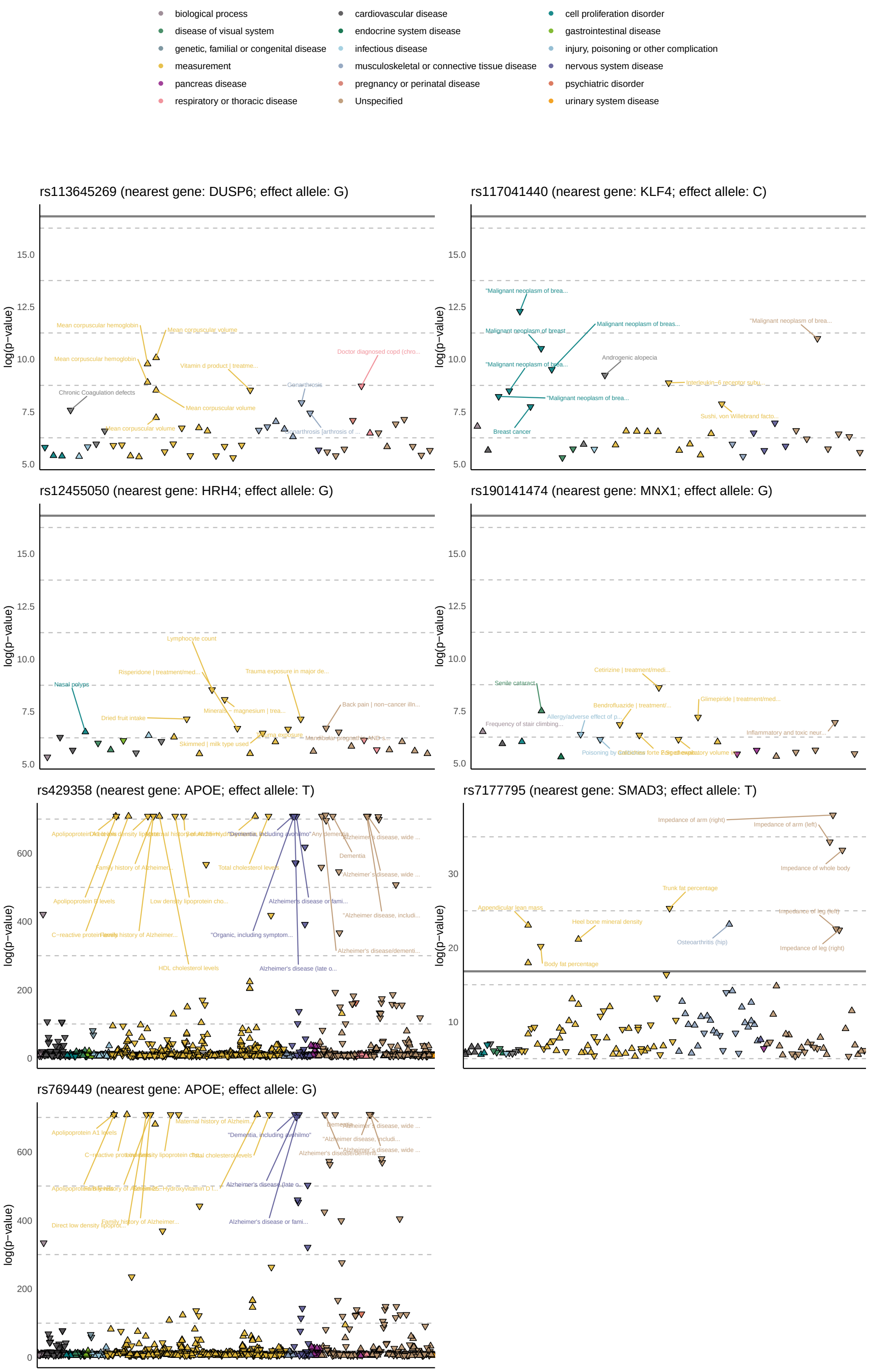

Supplementary Figure 9. Genotype-phenotype associations of variants associated with cognitive or physical decline . Lead SNPs were annotated to the nearest gene and mapped to previously associated phenotypes using the Open Target Genetics database, which curates summary statistic files from four sources: 1) GWAS analyses by NEALE (http://www.nealelab.is/uk-biobank) in the UK Biobank, 2) SAIGE analyses on binary phenotypes in the UKBB (https://www.leelabsg.org/resources), 3) the GWAS Catalog (GCST) (https://www.ebi.ac.uk/gwas/) and 4) FinnGen (https://www.finngen.fi/). The horizontal line in black represents the significant threshold ($P < 5 \times 10^{-8}$)

Supplementary Figure 10. Genotype-phenotype associations of variants showing sex-differential effects on physical function (composite score)

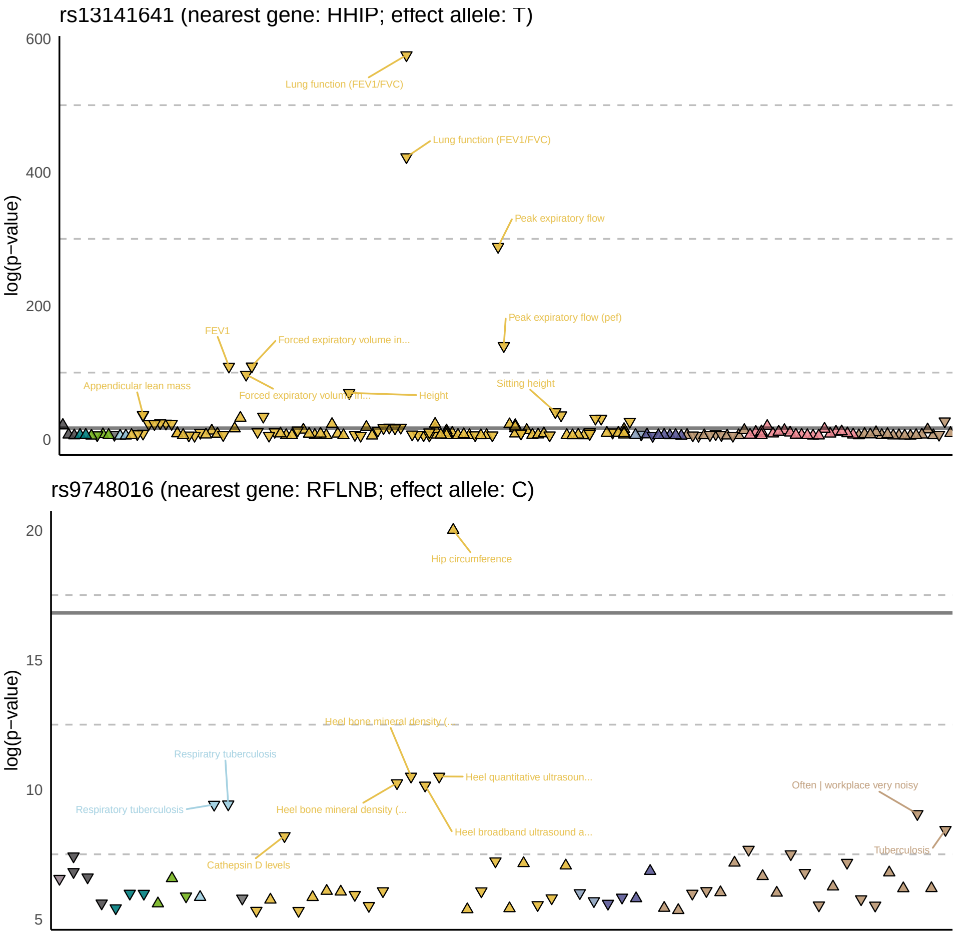

Supplementary Figure 10. Genotype-phenotype associations of variants showing sex-differential effects on physical function (composite s SNPs showing significant gene-by-sex interaction effects ($P < 5 \times 10^{-8}$) were annotated to the nearest gene and mapped to previously associated phenotypes using the Open Target Genetics database, which curates summary statistic files from four sources: 1) GWAS analyses by NEALE (http://www.nealelab.is/uk-biobank) in the UK Biobank, 2) SAIGE analyses on binary phenotypes in the UKBB (https://www.leelabsg.org/resources), 3) the GWAS Catalog (GCST) (https://www.ebi.ac.uk/gwas/) and 4) FinnGen (https://www.finngen.fi/). The horizontal line in black represents the significant threshold ($P < 5 \times 10^{-8}$)

Supplementary Figure 11. Weighted genome-wide analyses of variants associated with physical and cognitive outcomes

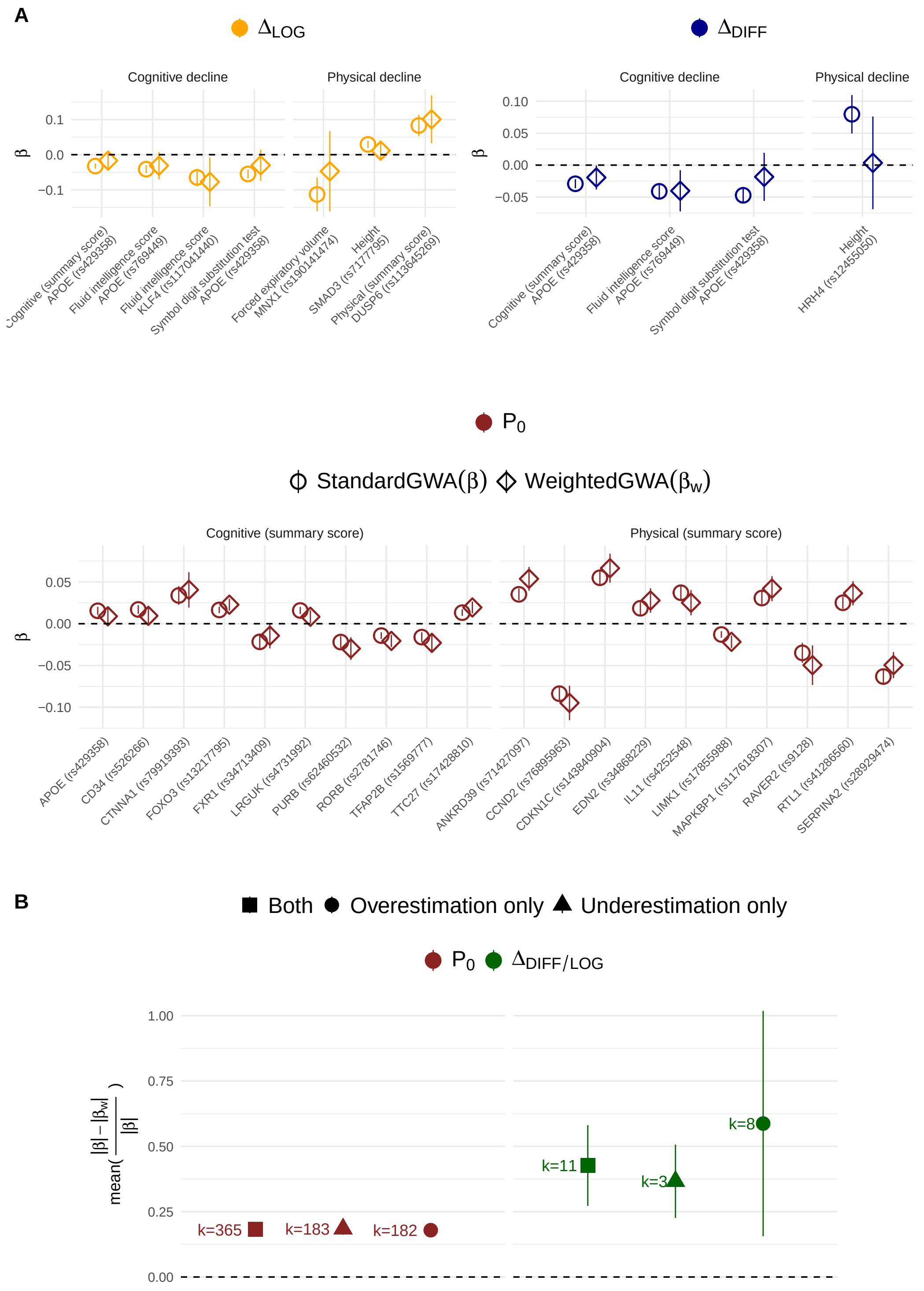

Supplementary Figure 11. Weighted genome-wide analyses of variants associated with physical and cognitive outcomes. **Panel A.** Standard and weighted variant effects were estimated using LDAK. We used the baseline sampling weights when performing weighted genome-wide scans on the baseline phenotypes (P_0) and the follow-up participation weights when testing variant effects on decline. Plotted are all effect estimates for variants significantly ($P < 5 \times 10^{-8}$) associated with change (Δ_{DIFF} and Δ_{LOG}), as well as variant effects that showed the largest abolute change ($|\hat{\beta}| - |\hat{\beta}_w|$) in genome-wide tests on baseline function (P_0). **Panel B.** The y-axis shows the mean proportional change in SNP effect estimates obtained from standard GWA (β) and weighted standard GWA (β_w) [$mean(\frac{|\beta|-|\beta_w|}{|\beta|})$]. k indexes the number of genetic variants included to obtain the mean proportional change, obtained from either all outcome-associated ($P < 5 \times 10^{-8}$) variants ('Both'), selected outcome-associated variants showing evidence of over-estimation ($|\hat{\beta}| - |\hat{\beta}_w| > 0$, 'Overestimation only') and selected outcome-associated variants showing evidence of under-estimation ($|\hat{\beta}| - |\hat{\beta}_w| < 0$, 'Underestimation only'). The full set of results is included in Supplementary Data 7.

Supplementary Figure 12. Predictors of decline obtained from phenotypic association tests and Mendelian Randomization analyses

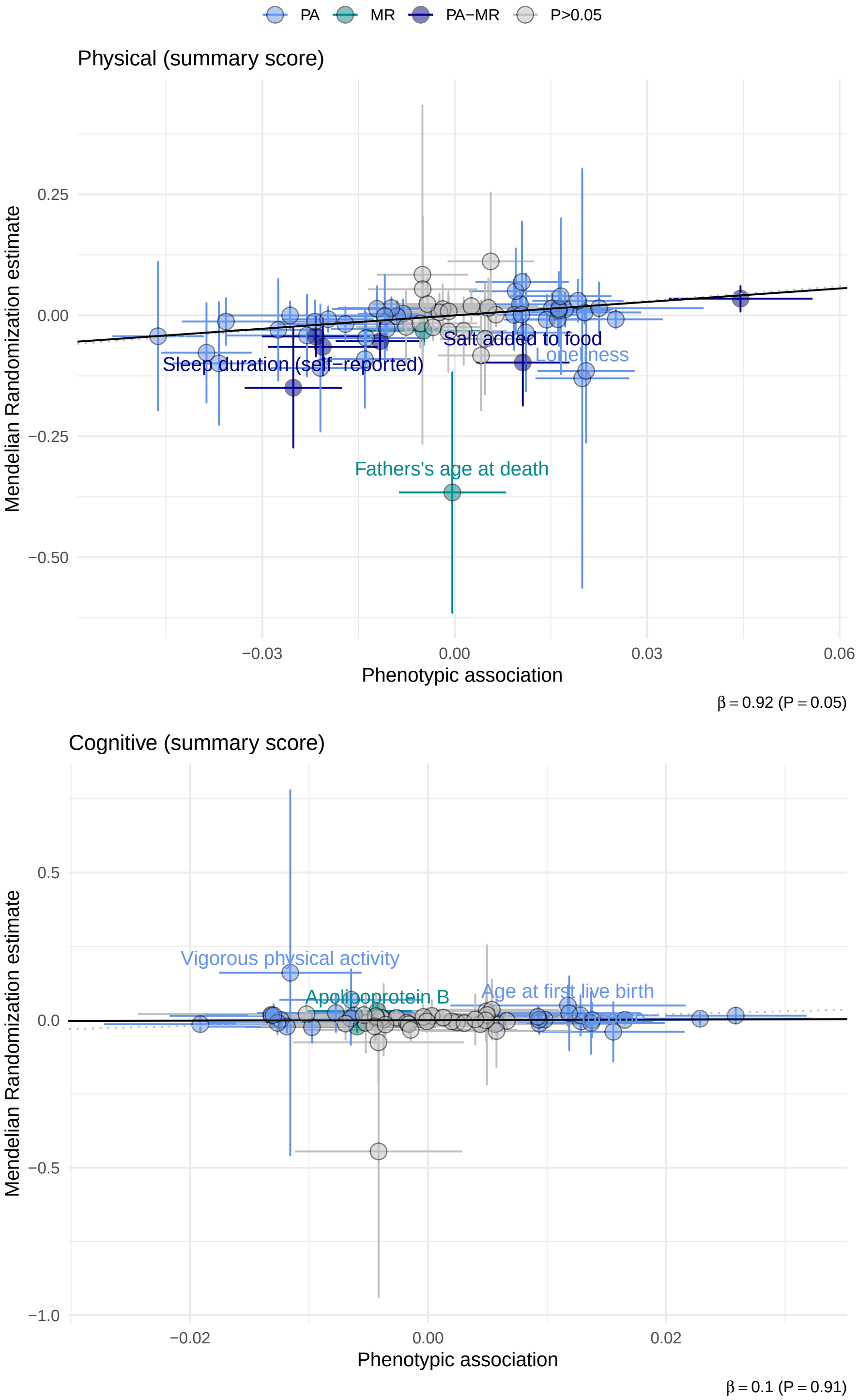

Supplementary Figure 12. Predictors of decline obtained from phenotypic association tests and Mendelian Randomization analyses. The standardized regression coefficients obtained from phenotypic tests of risk factors associated with change (defined as log-difference, Δ_{LOG}) are plotted against the standardized causal estimates obtained from Mendelian Randomization analyses. Excluded were exposures used to derive the summary scores of global cognitive and physical function. The legend indicates if a given estimate was identified (at $P < 0.05$) in phenotypic association tests only ('PA'), in Mendelian Randomization analysis only ('MA') or in both sets of analyses ('PA-MR'). Exposure-outcome associations that were not significant in either phenotypic or MR analyses are highlighted in grey. The black line and corresponding β-coefficient shown at the bottom of the figure provide the slope estimate of the line of best fit. All estimates obtained from phenotypic and MR analyses are provided in Supplementary Data 8.

Supplementary Figure 13. Predictors of cognitive decline (Mendelian Randomization)

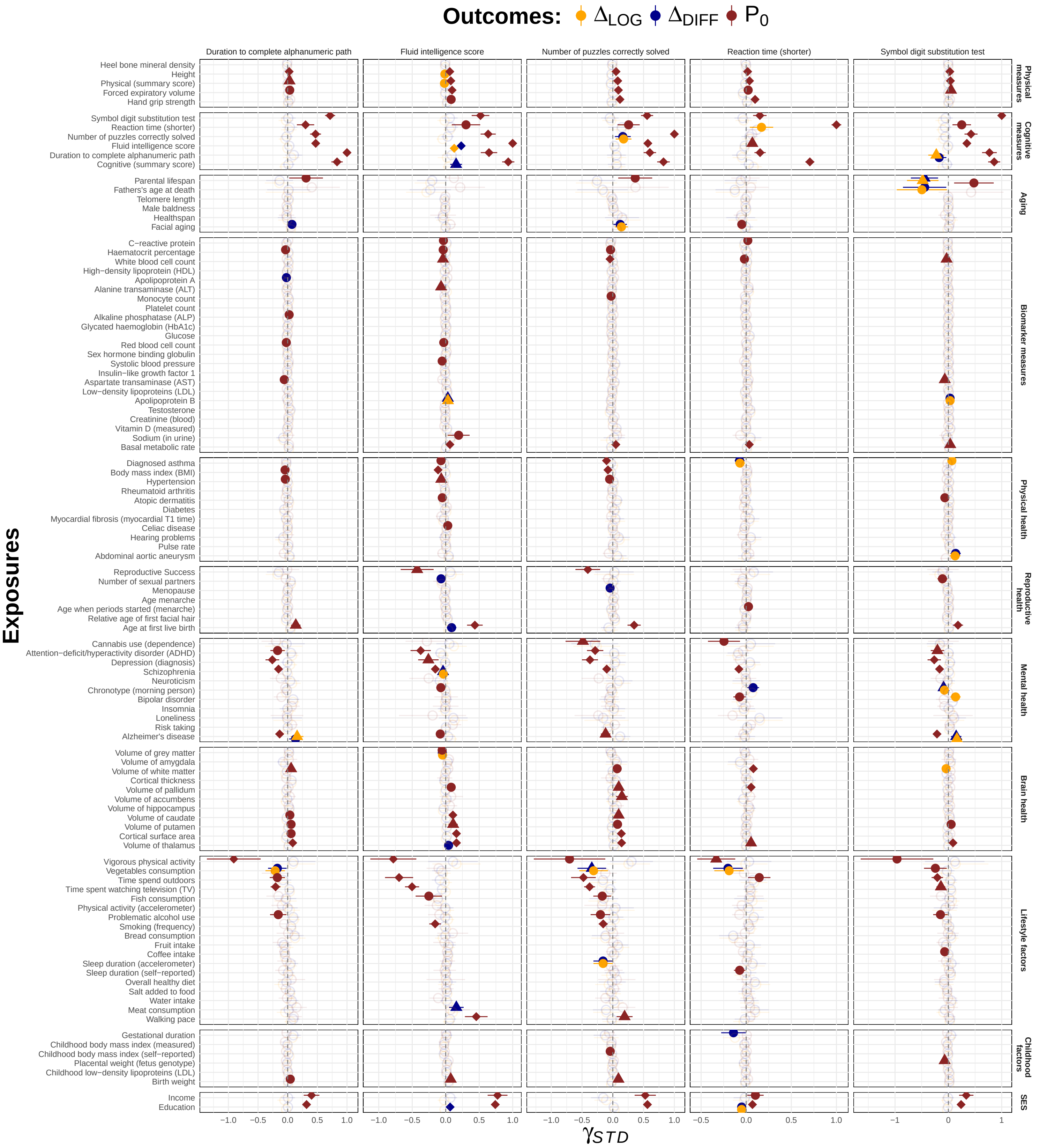

Supplementary Figure 13. Predictors of cognitive decline (Mendelian Randomization). Standardized Mendelian Randomization effect estimates (β_{STD}, with 95% confidence intervals) of exposure effects on cross-sectional outcomes (P_0 in red, with positive coefficients indexing higher levels of function) and longitudinal outcomes (Δ_{DIFF} in blue and Δ_{LOG} in orange, with positive coefficients indexing larger decline). Filled points, triangles and diamonds highlight significant effects (at $P < 0.05$, $P < 0.05/11$ and $P < 0.05/106$, respectively). Circles highlight non-significant ($P > 0.05$) effects.

Supplementary Figure 14. Predictors of physical decline (Mendelian Randomization)

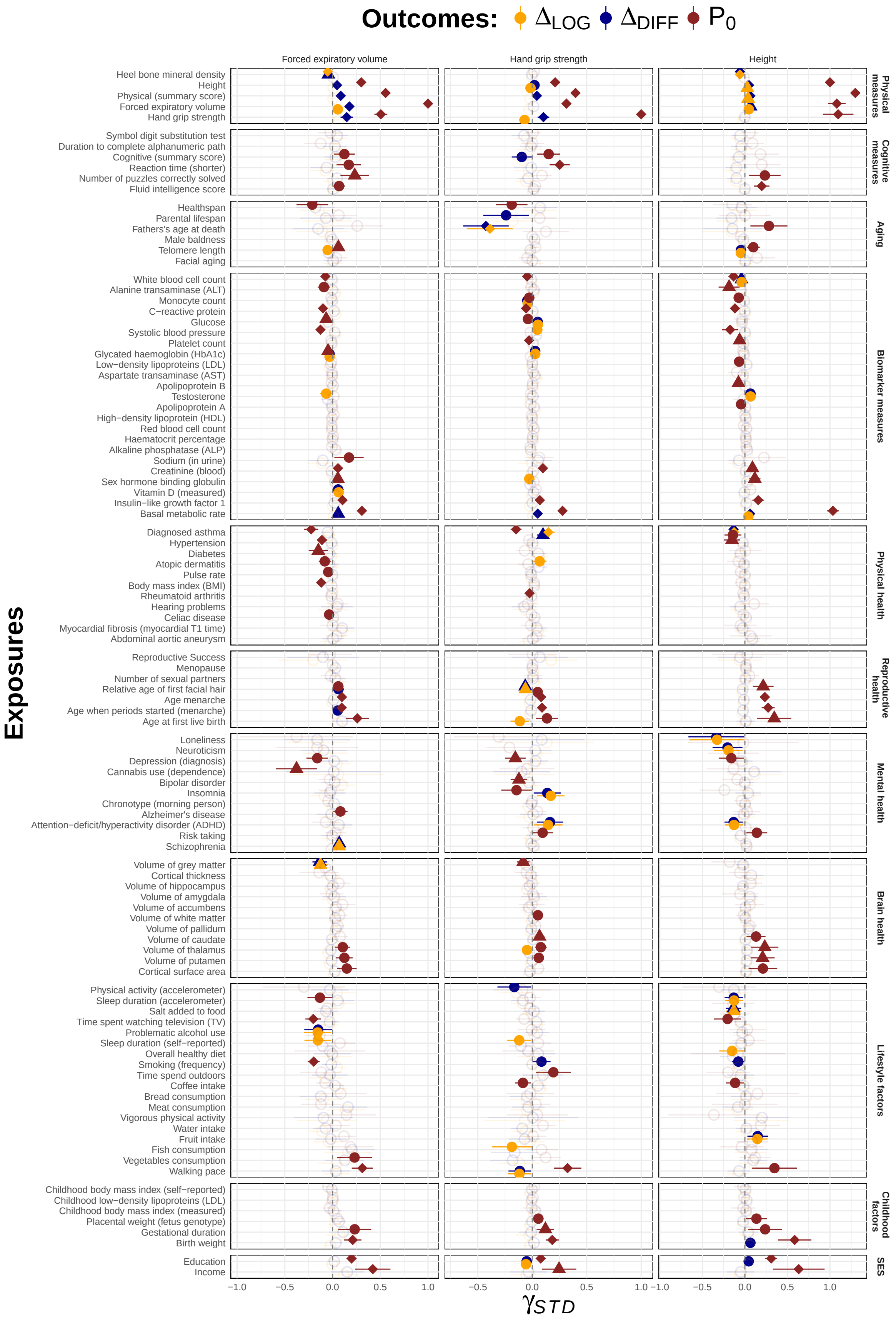

Supplementary Figure 14. Predictors of physical decline (Mendelian Randomization). Standardized Mendelian Randomization effect estimates (β_{STD}, with 95% confidence intervals) of exposure effects on cross-sectional outcomes (P_0 in red, with positive coefficients indexing higher levels of function) and longitudinal outcomes (Δ_{DIFF} in blue and Δ_{LOG} in orange, with positive coefficients indexing larger decline). Filled points, triangles and diamonds highlight significant effects (at $P < 0.05$, $P < 0.05/11$ and $P < 0.05/106$, respectively). Circles highlight non-significant ($P > 0.05$) effects.

Supplementary Figure 15. Longitudinal assessments of aging phenotypes in the UK Biobank

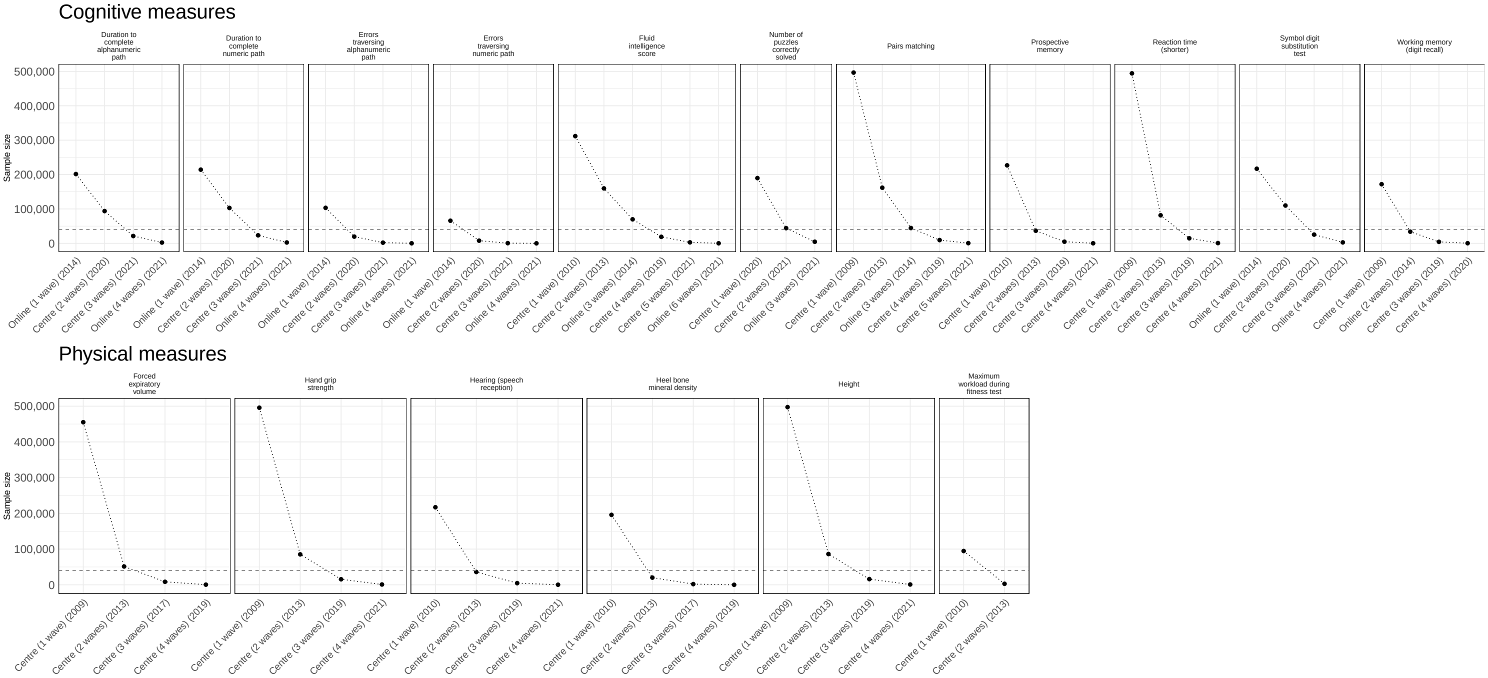

Supplementary Figure 15. Longitudinal assessments of aging phenotypes in the UK Biobank. Selected aging phenotypes used to derive indexes of physical and cognitive decline . The x-axis specifies the environment in which the phenotype was assessed at a given time point (i.e., assessment centre visit or online), the number of waves available for the phenotype and the mean year when the assessment took place per wave. The dashed line specifies the number individuals per phenotype with available 1-wave data (N with one assessment only), 2-wave data (N with at least two assessments), 3-wave data (N with at least three assessments), 4-wave data (N with at least four assessments). Included in genome-wide analyses were only phenotypes with at least 40,000 non-missing longitudinal (i.e., two-wave) observations (grey horizontal line).

Supplementary Figure 16. Correlations across measurement occasions for physical measures

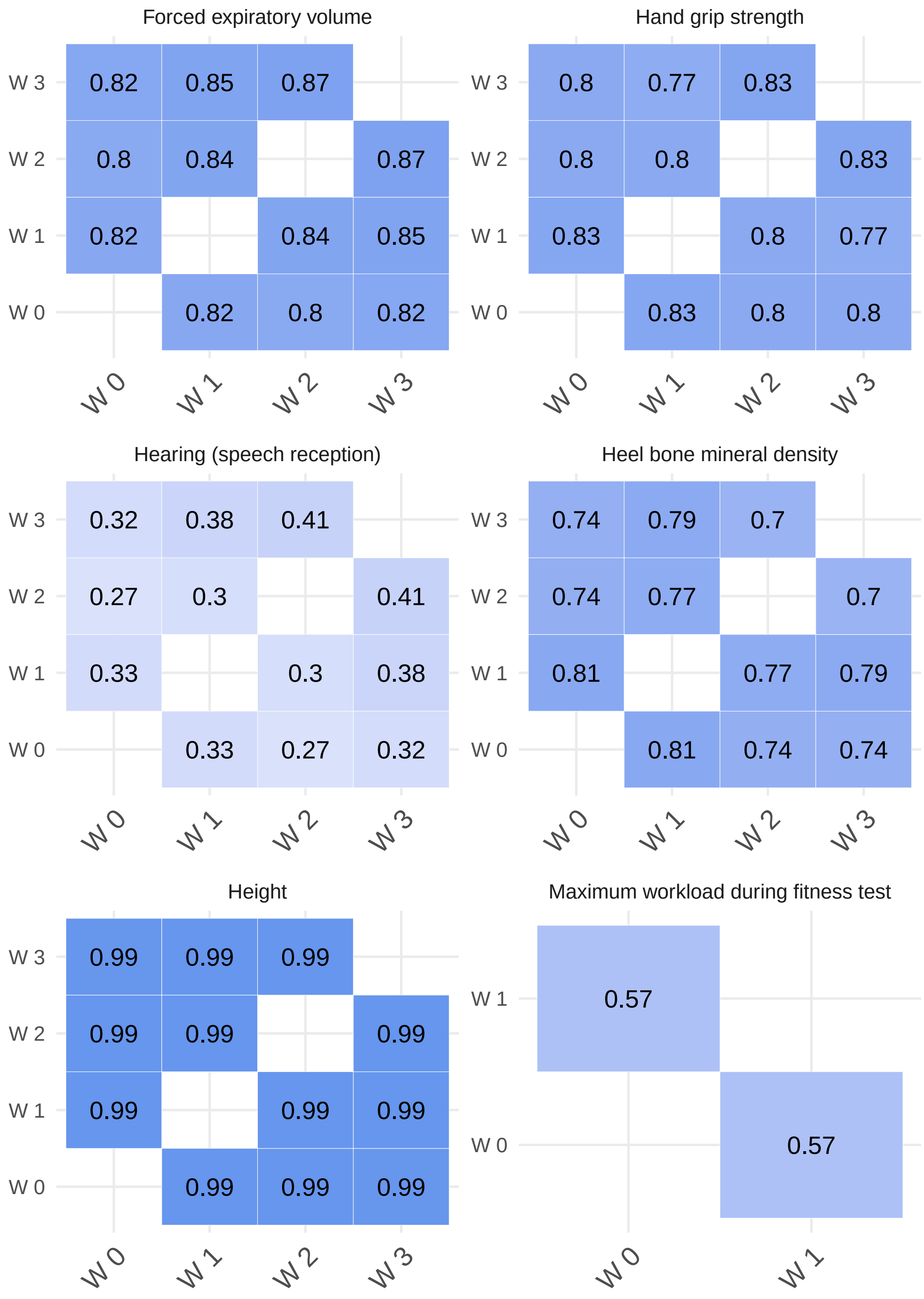

Supplementary Figure 16. Correlations across measurement occasions for physical measures. Mean correlation across measurement occasions for physical measures as follows: height: r =0.99; heel bone mineral density: r =0.76; forced expiratory volume: r =0.83; hearing (speech reception): r =0.33; maximum workload during fitness test: r =0.57; hand grip strength: r =0.81. Selected for global scores of physical decline were measures with a mean correlation > 0.4 across measurement occasions.

Supplementary Figure 17. Correlations across measurement occasions for cognitive measures

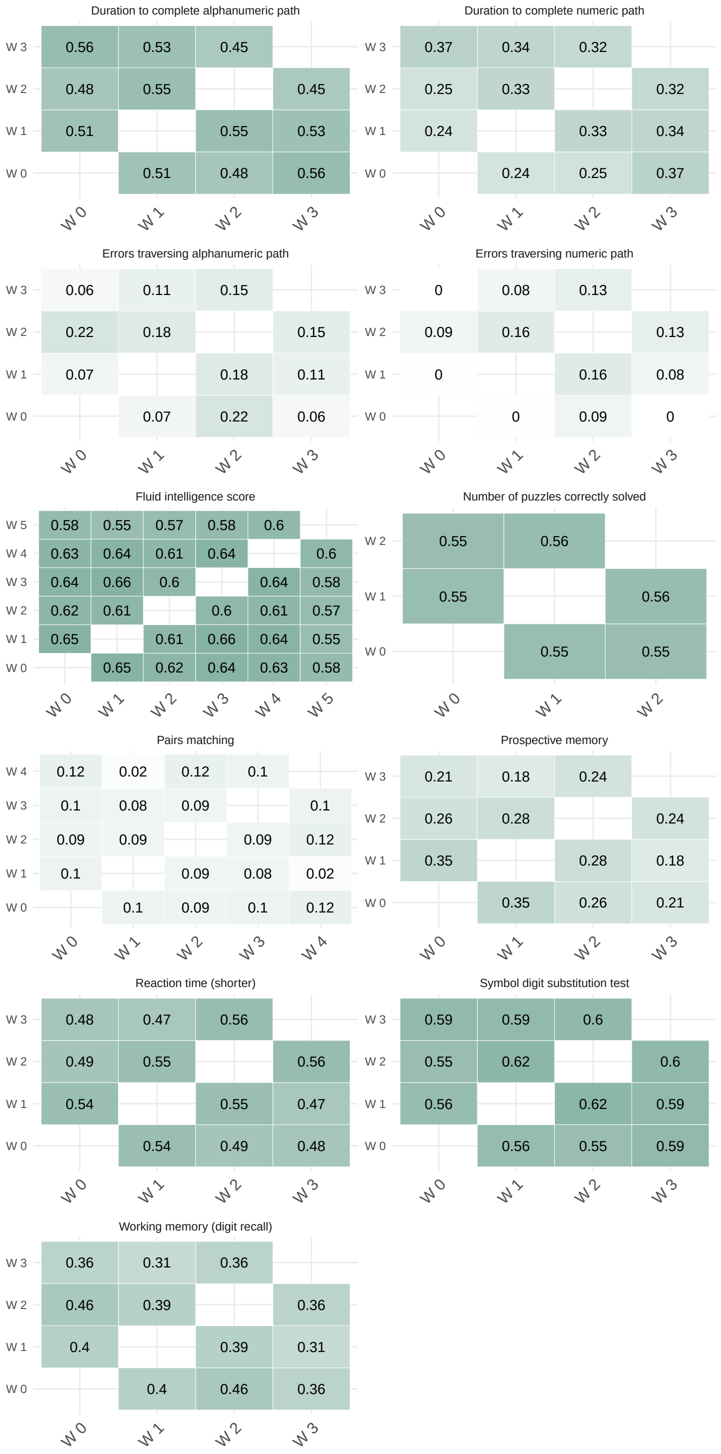

Supplementary Figure 17. Correlations across measurement occasions for cognitive measures. Mean correlation across measurement occasions for cognitive measures as follows: reaction time (shorter): r =0.52; prospective memory: r =0.25; working memory (digit recall): r =0.38; duration to complete numeric path: r =0.31; duration to complete alphanumeric path: r =0.51; errors traversing alphanumeric path: r =0.13; errors traversing numeric path: r =0.08; number of puzzles correctly solved: r =0.56; symbol digit substitution test: r =0.58; pairs matching: r =0.09; fluid intelligence score: r =0.61. Selected for global scores of cognitive decline were measures with a mean correlation > 0.4 across measurement occasions.

Supplementary Figure 18. Flow Diagram of the sample selection process

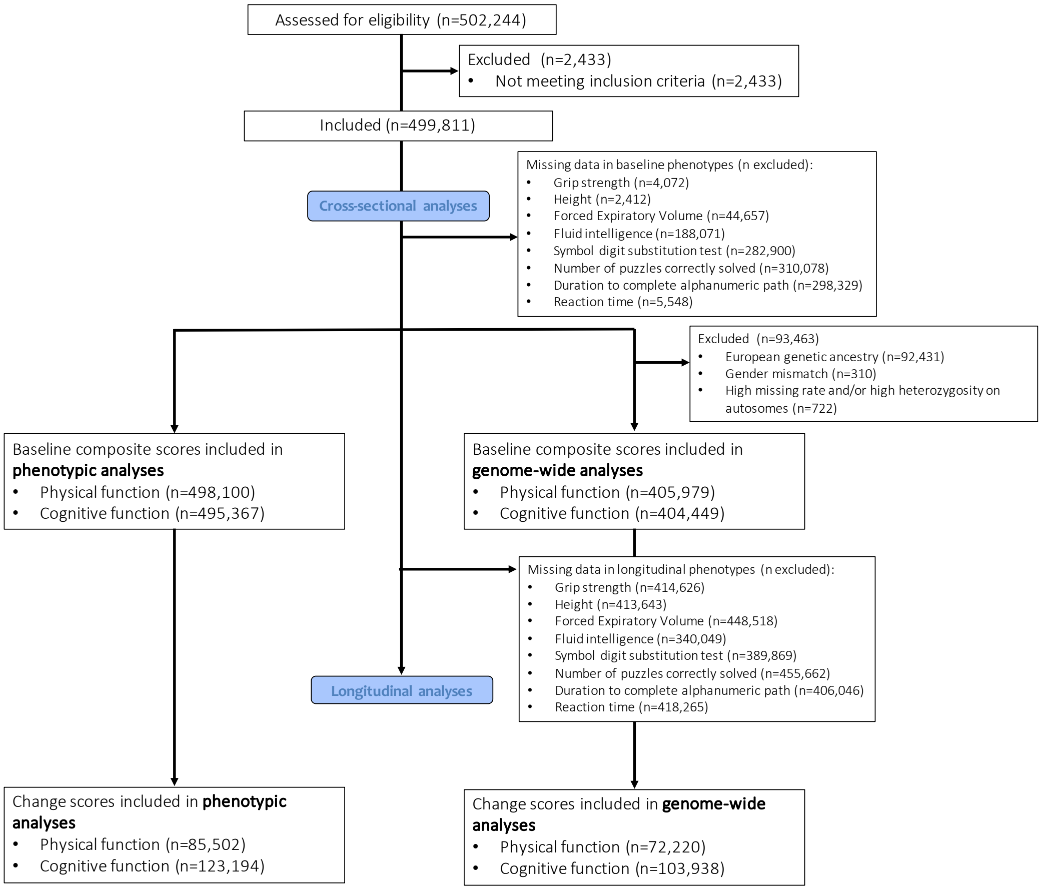

Supplementary Figure 18. Flow Diagram of the sample selection process. Selection of individuals included in phenotypic and genome-wide analyses.

Supplementary Figure 19. UK Biobank representativeness and characteristics of selective participation

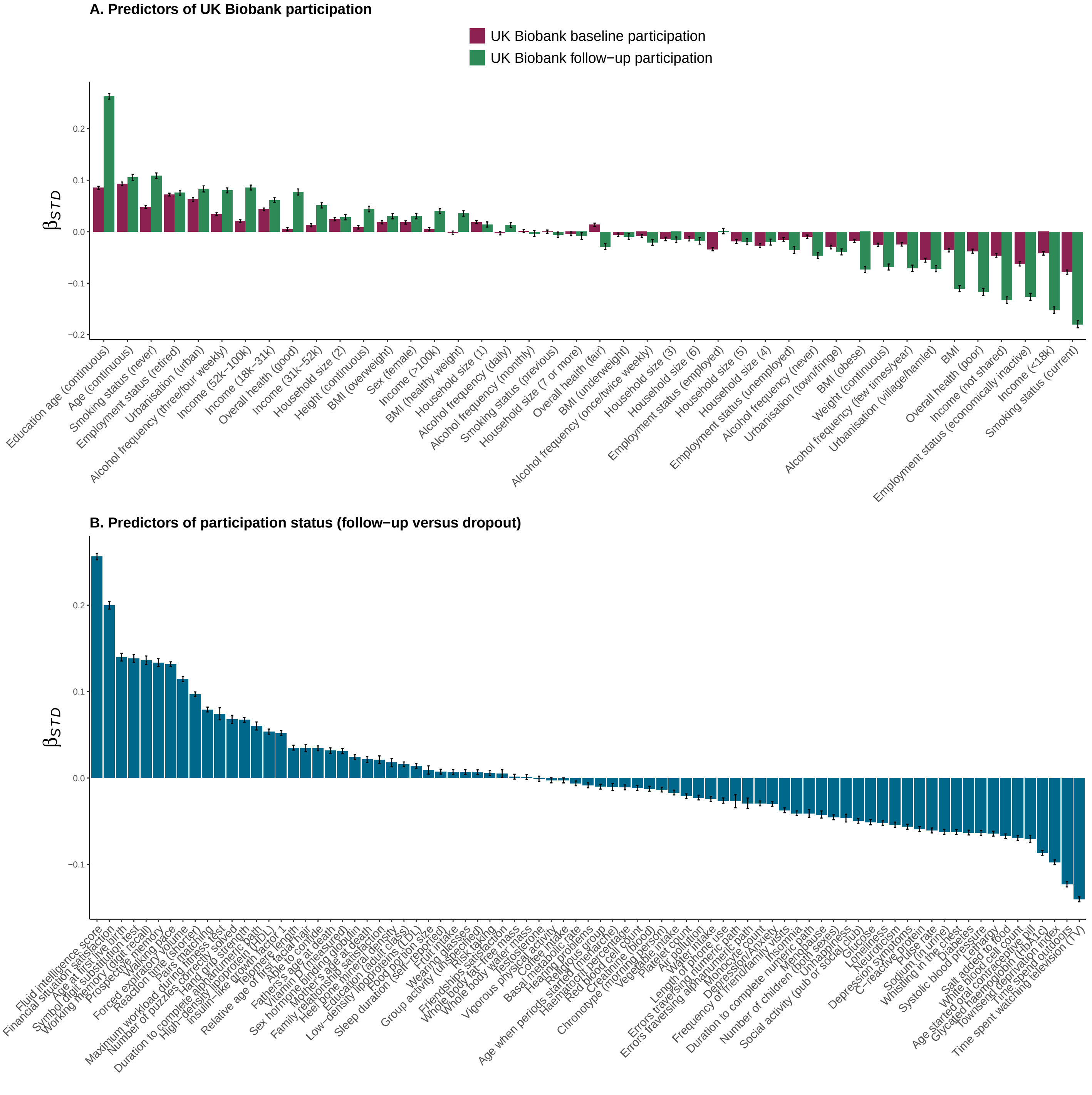

Supplementary Figure 19. UK Biobank representativeness and characteristics of selective participation. **Panel A.** Standardized β-coefficients of variables predicting UK Biobank baseline participation (in violet, Health Survey England=0; UKBB baseline sample=1) or follow-up participation (in green, Health Survey England=0; UKBB follow-up sample=1). **Panel B.** Standardized β-coefficients of variables predicting UKBB participation status (0=dropout; 1=included in follow-up). The error bars are the 95% confidence intervals.

Supplementary Figure 20. Genome-wide tests on cognitive and physical measures in the UK Biobank follow-up sample and dropout sample

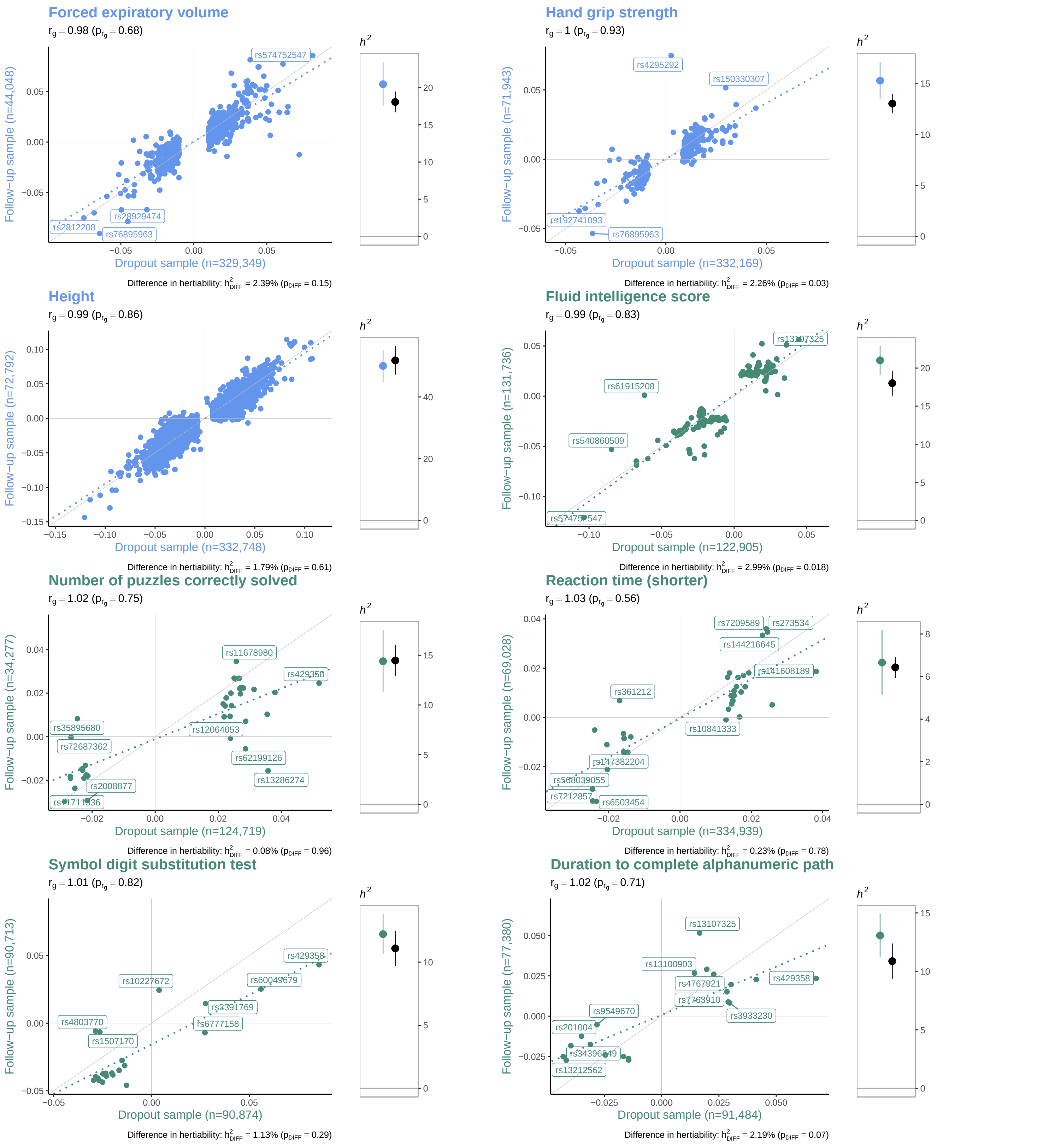

Supplementary Figure 20. Genome-wide tests on cognitive and physical measures in the UK Biobank follow-up sample and dropout sam The figure shows the effect estimates obtained from genome-wide tests on cognitive (in green) and physical (in blue) baseline measures, performed separately in the UK Biobank dropout sample (x-axis, S_D) and the follow-up sample (y-axis, S_{FU}). The dashed lines are the lines of best fit. r_g are the genetic correlation estimates obtained from LD-score regression to quantify the genetic relationship between S_D and S_{FU}. The corresponding P-values (P_{rg}) are obtained from Z-statistics ($Z = \frac{r_g - 1}{SE(r_g)}$) to test if r_g was significantly different from 1. The plotted dots highlight all SNP estimates reaching genome-wide significance ($P < 5 \times 10^{-8}$) in either the S_D or S_{FU}. The SNP-heritability estimates (h^2) obtained from the follow-up (in green or blue) and dropout sample (highlighted in black) are shown to the right of each panel.

Supplementary Figure 21. Distribution of baseline and follow−up participation weight

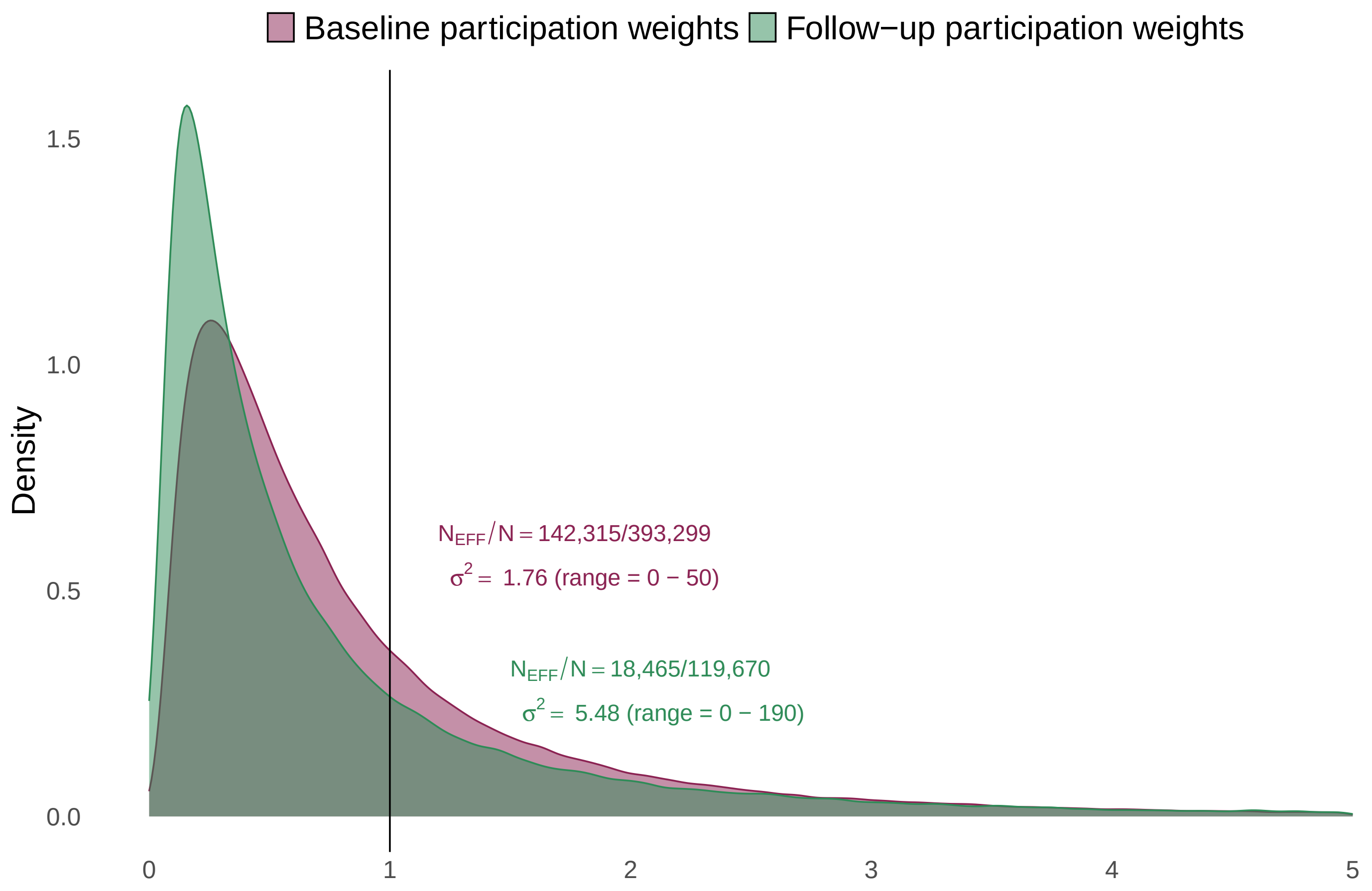

Supplementary Figure 21. Distribution of baseline and follow−up participation weight. The figure plots the truncated density curves of the probability weights (normalized to have a mean of one) to correct for selective baseline participation (in violet) and selective follow-up participation (in green). N = sample size of the unweighted UK Biobank sample; N_{EFF} = effective sample size of the weighted UK Biobank sample; σ^2 = variance of the sampling weights.

Supplementary References

1. Lyall, D. M. *et al.* Cognitive test scores in UK biobank: Data reduction in 480,416 participants and longitudinal stability in 20,346 participants. *PloS one* **11**, e0154222 (2016).
2. VanderWeele, T. J. *Explanation in causal inference: Methods for mediation and interaction.* (Oxford University Press, 2015).
3. Glymour, M. M., Weuve, J., Berkman, L. F., Kawachi, I. & Robins, J. M. When is baseline adjustment useful in analyses of change? An example with education and cognitive change. *American journal of epidemiology* **162**, 267–278 (2005).
4. Mindell, J. *et al.* Cohort profile: The health survey for england. *International journal of epidemiology* **41**, 1585–1593 (2012).
5. Schoeler, T. *et al.* Participation bias in the UK biobank distorts genetic associations and downstream analyses. *Nature Human Behaviour* **7**, 1216–1227 (2023).
6. Bulik-Sullivan, B. K. *et al.* LD score regression distinguishes confounding from polygenicity in genome-wide association studies. *Nature genetics* **47**, 291–295 (2015).
7. Lumley, T. *Complex surveys: A guide to analysis using r.* (John Wiley & Sons, 2011).
8. Zhang, Q., Privé, F., Vilhjálmsson, B. & Speed, D. Improved genetic prediction of complex traits from individual-level data or summary statistics. *Nature communications* **12**, 4192 (2021).
9. Trowman, R., Dumville, J. C., Torgerson, D. J. & Cranny, G. The impact of trial baseline imbalances should be considered in systematic reviews: A methodological case study. *Journal of clinical epidemiology* **60**, 1229–1233 (2007).
10. Oakes, J. M. & Feldman, H. A. Statistical power for nonequivalent pretest-posttest designs: The impact of change-score versus ANCOVA models. *Evaluation Review* **25**, 3–28 (2001).
11. Van Breukelen, G. J. ANCOVA versus change from baseline had more power in randomized studies and more bias in nonrandomized studies. *Journal of clinical epidemiology* **59**, 920–925 (2006).
12. Altman, D. G. Comparability of randomised groups. *Journal of the Royal Statistical Society Series D: The Statistician* **34**, 125–136 (1985).
13. Zhang, S. *et al.* Empirical comparison of four baseline covariate adjustment methods in analysis of continuous outcomes in randomized controlled trials. *Clinical epidemiology* 227–235 (2014).
14. Sadler, M. C. *et al.* Leveraging large-scale biobank EHRs to enhance pharmacogenetics of cardiometabolic disease medications. *medRxiv* (2024) doi:10.1101/2024.04.06.24305415.

15. Winkler, T. W. *et al.* Genetic-by-age interaction analyses on complex traits in UK biobank and their potential to identify effects on longitudinal trait change. *Genome Biology* **25**, 300 (2024).

16. Winkler, T. W. *et al.* The influence of age and sex on genetic associations with adult body size and shape: A large-scale genome-wide interaction study. *PLoS genetics* **11**, e1005378 (2015).

17. Ao, L. *et al.* Large-scale genome-wide interaction analyses on multiple cardiometabolic risk factors to identify age-specific genetic risk factors. *GeroScience* 1–13 (2024).

18. Chung, J. Y. *et al.* Identification of five genetic variants with differential effects on obesity-related traits based on age. *Frontiers in Genetics* **13**, 970657 (2022).

19. Simino, J. *et al.* Gene-age interactions in blood pressure regulation: A large-scale investigation with the CHARGE, global BPgen, and ICBP consortia. *The American Journal of Human Genetics* **95**, 24–38 (2014).

20. Winkler, T. W. *et al.* Investigating the modulation of genetic effects on late AMD by age and sex: Lessons learned and two additional loci. *PLoS One* **13**, e0194321 (2018).

21. Luan, J. *et al.* A multilevel linear mixed model of the association between candidate genes and weight and body mass index using the framingham longitudinal family data. in *BMC proceedings* vol. 3 1–6 (Springer, 2009).

22. Bretsky, P., Guralnik, J., Launer, L., Albert, M. & Seeman, T. The role of APOE-$\varepsilon 4$ in longitudinal cognitive decline: MacArthur studies of successful aging. *Neurology* **60**, 1077–1081 (2003).

23. Roslin, N. M., Hamid, J. S., Paterson, A. D. & Beyene, J. Genome-wide association analysis of cardiovascular-related quantitative traits in the framingham heart study. in *BMC proceedings* vol. 3 1–6 (Springer, 2009).

24. Allen, R. J. *et al.* Longitudinal lung function and gas transfer in individuals with idiopathic pulmonary fibrosis: A genome-wide association study. *The Lancet Respiratory Medicine* **11**, 65–73 (2023).

25. De Jager, P. L. *et al.* A genome-wide scan for common variants affecting the rate of age-related cognitive decline. *Neurobiology of aging* **33**, 1017–e1 (2012).

26. Kamboh, M. I. *et al.* Population-based genome-wide association study of cognitive decline in older adults free of dementia: Identification of a novel locus for the attention domain. *Neurobiology of aging* **84**, 239–e15 (2019).

27. Wendel, B. *et al.* A genome-wide association study of the longitudinal course of executive functions. *Translational Psychiatry* **11**, 386 (2021).

28. Venkatesh, S. S. *et al.* Characterising the genetic architecture of changes in adiposity during adulthood using electronic health records. *Nature Communications* **15**, 5801 (2024).

29. Ko, S. *et al.* GWAS of longitudinal trajectories at biobank scale. *The American Journal of Human Genetics* **109**, 433–445 (2022).

30. Gow, A. J. *et al.* Is age kinder to the initially more able?: Yes, and no. *Intelligence* **40**, 49–59 (2012).

31. Christensen, H. & Henderson, A. Is age kinder to the initially more able? A study of eminent scientists and academics. *Psychological medicine* **21**, 935–946 (1991).

32. Deary, I. J., Starr, J. M. & MacLennan, W. J. Is age kinder to the initially more able?: Differential ageing of a verbal ability in the healthy old people in edinburgh study. *Intelligence* **26**, 357–375 (1998).

33. Stern, Y. What is cognitive reserve? Theory and research application of the reserve concept. *Journal of the international neuropsychological society* **8**, 448–460 (2002).

34. Stern, Y. *et al.* Whitepaper: Defining and investigating cognitive reserve, brain reserve, and brain maintenance. *Alzheimer's & Dementia* **16**, 1305–1311 (2020).

35. Kuh, D., Karunananthan, S., Bergman, H. & Cooper, R. A life-course approach to healthy ageing: Maintaining physical capability. *Proceedings of the Nutrition Society* **73**, 237–248 (2014).

36. Salthouse, T. A. Mental exercise and mental aging: Evaluating the validity of the 'use it or lose it' hypothesis. *Perspectives on psychological science* **1**, 68–87 (2006).

37. Bosma, H., Van Boxtel, M., Ponds, R., Houx, P. & Jolles, J. Education and age-related cognitive decline: The contribution of mental workload. *Educational gerontology* **29**, 165–173 (2003).

38. Lyketsos, C. G., Chen, L.-S. & Anthony, J. C. Cognitive decline in adulthood: An 11.5-year follow-up of the baltimore epidemiologic catchment area study. *American Journal of Psychiatry* **156**, 58–65 (1999).

39. Teri, L., McCurry, S. M., Edland, S. D., Kukull, W. A. & Larson, E. B. Cognitive decline in alzheimer's disease: A longitudinal investigation of risk factors for accelerated decline. *The Journals of Gerontology Series A: Biological Sciences and Medical Sciences* **50**, M49–M55 (1995).

40. Richards, M., Shipley, B., Fuhrer, R. & Wadsworth, M. E. Cognitive ability in childhood and cognitive decline in mid-life: Longitudinal birth cohort study. *Bmj* **328**, 552 (2004).

41. Fjell, A. M. *et al.* Reevaluating the role of education in cognitive decline and brain aging: Insights from large-scale longitudinal cohorts across 33 countries. *medRxiv* (2025) doi:10.1101/2025.01.29.25321305.

42. Tucker-Drob, E. M., Johnson, K. E. & Jones, R. N. The cognitive reserve hypothesis: A longitudinal examination of age-associated declines in reasoning and processing speed. *Developmental psychology* **45**, 431 (2009).

43. Ritchie, S. J., Tucker-Drob, E. M., Starr, J. M. & Deary, I. J. Do cognitive and physical functions age in concert from age 70 to 76? Evidence from the lothian birth cohort 1936. *The Spanish Journal of Psychology* **19**, E90 (2016).

44. Zahodne, L. B. *et al.* Education does not slow cognitive decline with aging: 12-year evidence from the victoria longitudinal study. *Journal of the International Neuropsychological Society* **17**, 1039–1046 (2011).

45. Singh-Manoux, A. *et al.* Does cognitive reserve shape cognitive decline? *Annals of neurology* **70**, 296–304 (2011).

46. Christensen, H. *et al.* Age is no kinder to the better educated: Absence of an association investigated using latent growth techniques in a community sample. *Psychological medicine* **31**, 15–28 (2001).

47. Piccinin, A. M. *et al.* Coordinated analysis of age, sex, and education effects on change in MMSE scores. *Journals of Gerontology Series B: Psychological Sciences and Social Sciences* **68**, 374–390 (2013).

48. Gottesman, R. F. *et al.* Impact of differential attrition on the association of education with cognitive change over 20 years of follow-up: The ARIC neurocognitive study. *American journal of epidemiology* **179**, 956–966 (2014).

49. Clouston, S. A., Glymour, M. M. & Terrera, G. M. Educational inequalities in aging-related declines in fluid cognition and the onset of cognitive pathology. *Alzheimer's & Dementia: Diagnosis, Assessment & Disease Monitoring* **1**, 303–310 (2015).

50. Clouston, S. A. *et al.* The dynamic relationship between physical function and cognition in longitudinal aging cohorts. *Epidemiologic reviews* **35**, 33–50 (2013).

51. Christensen, H. *et al.* Are changes in sensory disability, reaction time, and grip strength associated with changes in memory and crystallized intelligence? A longitudinal analysis in an elderly community sample. *Gerontology* **46**, 276–292 (2000).

52. Baltes, P. B. & Lindenberger, U. Emergence of a powerful connection between sensory and cognitive functions across the adult life span: A new window to the study of cognitive aging? *Psychology and aging* **12**, 12 (1997).

53. Zammit, A. R., Robitaille, A., Piccinin, A. M., Muniz-Terrera, G. & Hofer, S. M. Associations between aging-related changes in grip strength and cognitive function in older adults: A systematic review. *The Journals of Gerontology: Series A* **74**, 519–527 (2019).

54. Deary, I. J. *et al.* Losing one's grip: A bivariate growth curve model of grip strength and nonverbal reasoning from age 79 to 87 years in the lothian birth cohort 1921. *Journals of Gerontology Series B: Psychological Sciences and Social Sciences* **66**, 699–707 (2011).

55. Hofer, S. M., Berg, S. & Era, P. Evaluating the interdependence of aging-related changes in visual and auditory acuity, balance, and cognitive functioning. *Psychology and aging* **18**, 285 (2003).

56. Ritchie, S. J. *et al.* Predictors of ageing-related decline across multiple cognitive functions. *Intelligence* **59**, 115–126 (2016).

57. Yaffe, K. *et al.* Cardiovascular risk factors across the life course and cognitive decline: A pooled cohort study. *Neurology* **96**, e2212–e2219 (2021).

58. Xu, Y. *et al.* Cumulative blood pressure load and cognitive decline in older adults: An observational analysis of two large cohorts. *Cerebral Circulation-Cognition and Behavior* 100375 (2024).

59. Song, F. *et al.* Plasma apolipoprotein levels are associated with cognitive status and decline in a community cohort of older individuals. *PloS one* **7**, e34078 (2012).

60. Fletcher, E. *et al.* Brain volume change and cognitive trajectories in aging. *Neuropsychology* **32**, 436 (2018).

61. Blondell, S. J., Hammersley-Mather, R. & Veerman, J. L. Does physical activity prevent cognitive decline and dementia?: A systematic review and meta-analysis of longitudinal studies. *BMC public health* **14**, 1–12 (2014).

62. Iso-Markku, P. *et al.* Physical activity and cognitive decline among older adults: A systematic review and meta-analysis. *JAMA network open* **7**, e2354285–e2354285 (2024).

63. Sabia, S. *et al.* Alcohol consumption and cognitive decline in early old age. *Neurology* **82**, 332–339 (2014).

64. Topiwala, A. *et al.* Moderate alcohol consumption as risk factor for adverse brain outcomes and cognitive decline: Longitudinal cohort study. *bmj* **357**, (2017).

65. Sabia, S. *et al.* Impact of smoking on cognitive decline in early old age: The whitehall II cohort study. *Archives of general psychiatry* **69**, 627–635 (2012).

66. Loef, M. & Walach, H. Fruit, vegetables and prevention of cognitive decline or dementia: A systematic review of cohort studies. *The Journal of nutrition, health and aging* **16**, 626–630 (2012).

67. Brenowitz, W. D. *et al.* Depressive symptoms imputed across the life course are associated with cognitive impairment and cognitive decline. *Journal of Alzheimer's Disease* **83**, 1379–1389 (2021).

68. Penninx, B. W., Deeg, D. J., Van Eijk, J. T. M., Beekman, A. T. & Guralnik, J. M. Changes in depression and physical decline in older adults: A longitudinal perspective. *Journal of affective disorders* **61**, 1–12 (2000).

69. Mander, B. A., Winer, J. R. & Walker, M. P. Sleep and human aging. *Neuron* **94**, 19–36 (2017).

70. Spira, A. P. *et al.* Poor sleep quality and functional decline in older women. *Journal of the American Geriatrics Society* **60**, 1092–1098 (2012).

71. Krishna, M. *et al.* Size at birth and cognitive ability in late life: A systematic review. *International Journal of Geriatric Psychiatry* **34**, 1139–1169 (2019).

72. Pollak, C., Verghese, J. & Blumen, H. Loneliness and functional decline in aging: A systematic review. *Research in gerontological nursing* **16**, 202–212 (2023).

73. Steptoe, A. & Zaninotto, P. Lower socioeconomic status and the acceleration of aging: An outcome-wide analysis. *Proceedings of the National Academy of Sciences* **117**, 14911–14917 (2020).

74. Carrillo, J. Á., Arcusa, R., Zafrilla, M. P. & Marhuenda, J. Effects of fruit and vegetable-based nutraceutical on cognitive function in a healthy population: Placebo-controlled, double-blind, and randomized clinical trial. *Antioxidants* **10**, 116 (2021).

75. Forte, P. *et al.* The effects of an 8-month multicomponent training program in body composition, functional fitness, and sleep quality in aged people: A randomized controlled trial. *Journal of Clinical Medicine* **13**, 6603 (2024).

76. Thalén, A. & Ledberg, A. Consequences of heterogeneity in aging: Parental age at death predicts midlife all-cause mortality and hospitalization in a swedish national birth cohort. *BMC geriatrics* **24**, 207 (2024).

77. Shadyab, A. H. *et al.* Parental longevity predicts healthy ageing among women. *Age and Ageing* **47**, 853–860 (2018).

78. Dutta, A. *et al.* Aging children of long-lived parents experience slower cognitive decline. *Alzheimer's & Dementia* **10**, S315–S322 (2014).

79. Aschard, H. A perspective on interaction effects in genetic association studies. *Genetic epidemiology* **40**, 678–688 (2016).

80. Chandra, M. *et al.* Air pollution and cognitive impairment across the life course in humans: A systematic review with specific focus on income level of study area. *International journal of environmental research and public health* **19**, 1405 (2022).

81. Hanes, D. W. & Clouston, S. A. Cognitive decline after divorce and widowhood: Is marital loss always a loss? *Innovation in Aging* **8**, igae033 (2024).

82. Tschanz, J. T. *et al.* Stressful life events and cognitive decline in late life: Moderation by education and age. The cache county study. *International journal of geriatric psychiatry* **28**, 821–830 (2013).

83. Kim, M. H. & Schwartz, G. L. Foreclosure, memory decline, and dementia probability: A longitudinal cohort study. *Alzheimer's & dementia* **20**, 6517–6526 (2024).
