# Supplementary material for: Combining cross-sectional and longitudinal genomic approaches to identify determinants of cognitive and physical decline
Source: Nat Commun. 2025 May 15;16:4524. doi: 10.1038/s41467-025-59383-0 (PMC12081661; doi:10.1038/s41467-025-59383-0)
Supplement: Supplementary file 2 — Description of Additional Supplementary Files [file 41467_2025_59383_MOESM2_ESM.pdf]

Description of Additional Supplementary Files

Supplementary Data 1. Age effects on aging phenotypes across levels of sample representativeness

Age effects (beta, with with 95% confidence intervals) on cognitive and physical measures were obtained from linear regression models, applied in a) the unweighted UKBB baseline sample ('baseline sample, 1−wave'), b) the inverse probability weighted UKBB baseline sample ('weighted 1−wave'), c) the UKBB follow-up sample with complete data in at least one follow-up assessment ('follow−up sample, 2−waves') and d) the UKBB follow-up sample with complete data in at least two follow-up assessments ('follow−up sample, 3−waves'). Phenotypes shown in this table include those with little missing data when assessed at baseline (i.e., at least 450,000 UKBB participants). The negative beta-coefficient represents decline in standard deviations in the outcome per additional year of age.

Supplementary Data 2. Follow-up duration and sample size per longitudinal aging phenotype

Supplementary Data 3. Cross-sectional and longitudinal genetic variant effects

Source Data corresponding to the results shown in Figure 4 (main manuscript) and Supplementary Figure 8-9. The column 'outcome type' specifies the model that was used to derive the outcome phenotype, including 1) log = log-difference model (relative change), diff = difference score (absolute change) 3) res = residual change score model (conditional change) and 4) baseline = the cross-sectional phenotype assessed at baseline.

Supplementary Data 4. Mendelian Randomization results

Source Data corresponding to the results shown in Figure 5 (main manuscript) and Supplementary Figure 13-14. The column 'outcome type' specifies the model that was used to derive the outcome phenotype, including 1) log = log-difference model (relative change), diff = difference score (absolute change) 3) res = residual change score model (conditional change) and 4) baseline = the cross-sectional phenotype assessed at baseline.

Supplementary Data 5. Missing data and dates of follow-up for cognitive and physical variables

Source Data corresponding to the results shown in Supplementary Figure 15.

Supplementary Data 6. Summary statistic files included in Mendelian Randomization analyses

Supplementary Data 7. Standard and Inverse Probability Weighted genetic variant effects

Source Data corresponding to the results shown and Supplementary Figure 11. The column 'outcome type' specifies the model that was used to derive the outcome phenotype, including 1) log = log-difference model (relative change), diff = difference score (absolute change) 3) res = residual change score model (conditional change) and 4) baseline = the cross-sectional phenotype assessed at baseline.

Supplementary Data 8. Phenotypic associations with change (log-difference)

Source Data corresponding to the results shown and Supplementary Figure 12. Standardized regression coefficients (beta, with with 95% confidence intervals) obtained from phenotypic analyses of risk factors associated with change (defined as log-difference).
