# Supplementary material for: Combining cross-sectional and longitudinal genomic approaches to identify determinants of cognitive and physical decline
Source: Nat Commun. 2025 May 15;16:4524. doi: 10.1038/s41467-025-59383-0 (PMC12081661; doi:10.1038/s41467-025-59383-0)
Supplement: Supplementary file 4 — Reporting Summary [file 41467_2025_59383_MOESM4_ESM.pdf]

nature portfolio

Corresponding author(s): Tabea Schoeler, Zoltán Kutalik

Last updated by author(s): Apr 9, 2025

Reporting Summary

Nature Portfolio wishes to improve the reproducibility of the work that we publish. This form provides structure for consistency and transparency in reporting. For further information on Nature Portfolio policies, see our Editorial Policies and the Editorial Policy Checklist.

Statistics

For all statistical analyses, confirm that the following items are present in the figure legend, table legend, main text, or Methods section.

n/a	Confirmed	
☐	☒	The exact sample size (*n*) for each experimental group/condition, given as a discrete number and unit of measurement
☐	☒	A statement on whether measurements were taken from distinct samples or whether the same sample was measured repeatedly
☐	☒	The statistical test(s) used AND whether they are one- or two-sided<br>*Only common tests should be described solely by name; describe more complex techniques in the Methods section.*
☐	☒	A description of all covariates tested
☐	☒	A description of any assumptions or corrections, such as tests of normality and adjustment for multiple comparisons
☐	☒	A full description of the statistical parameters including central tendency (e.g. means) or other basic estimates (e.g. regression coefficient) AND variation (e.g. standard deviation) or associated estimates of uncertainty (e.g. confidence intervals)
☐	☒	For null hypothesis testing, the test statistic (e.g. *F*, *t*, *r*) with confidence intervals, effect sizes, degrees of freedom and *P* value noted<br>*Give P values as exact values whenever suitable.*
☒	☐	For Bayesian analysis, information on the choice of priors and Markov chain Monte Carlo settings
☒	☐	For hierarchical and complex designs, identification of the appropriate level for tests and full reporting of outcomes
☐	☒	Estimates of effect sizes (e.g. Cohen's *d*, Pearson's *r*), indicating how they were calculated

Our web collection on statistics for biologists contains articles on many of the points above.

Software and code

Policy information about availability of computer code

Data collection	This research has been conducted with the UK Biobank Resource under application number 16389.
Data analysis	The following software was used to run the analyses: REGENIE (https://github.com/rgcgithub/regenie); TwoSampleMR (https://mrcieu.github.io/TwoSampleMR/); GenomicSEM (https://github.com/GenomicSEM/GenomicSEM); otargen (https://amirfeizi.github.io/otargen/); LDAK (https://dougspeed.com/). All analytical scripts are available at https://github.com/TabeaSchoeler/TS2024_UKBBlongitudinal

For manuscripts utilizing custom algorithms or software that are central to the research but not yet described in published literature, software must be made available to editors and reviewers. We strongly encourage code deposition in a community repository (e.g. GitHub). See the Nature Portfolio guidelines for submitting code & software for further information.

Data

Policy information about availability of data

All manuscripts must include a data availability statement. This statement should provide the following information, where applicable:

- Accession codes, unique identifiers, or web links for publicly available datasets
- A description of any restrictions on data availability
- For clinical datasets or third party data, please ensure that the statement adheres to our policy

Provide your data availability statement here.

Research involving human participants, their data, or biological material

Policy information about studies with human participants or human data. See also policy information about sex, gender (identity/presentation), and sexual orientation and race, ethnicity and racism.

Reporting on sex and gender	We used self-reported sex (biological attribute) in our study.
Reporting on race, ethnicity, or other socially relevant groupings	NA
Population characteristics	In genome-wide analyses, we included UK Biobank participants of European ancestry passing standard GWA analysis quality control measures. All analyses were adjusted for batch, principal components, age and sex. Exclusions during QC process (phenotypic and genetic) are detailed in the Methods.
Recruitment	The UK Biobank (UKBB) is a prospective population-based research resource focusing on the role of genetic, environmental and lifestyle factors in health outcomes in middle age and later life. More than 9,000,000 men and women between 40 and 69 registered with the UK NHS were invited to take part. Of those, 5.4% (~500,000 individuals) were recruited in 22 assessment centres across England, Wales and Scotland between 2006 and 2010.
Ethics oversight	The UK Biobank resource was approved by the UK Biobank Research Ethics Committee and all participants provided written informed consent to participate.

Note that full information on the approval of the study protocol must also be provided in the manuscript.

Field-specific reporting

Please select the one below that is the best fit for your research. If you are not sure, read the appropriate sections before making your selection.

☒ Life sciences ☐ Behavioural & social sciences ☐ Ecological, evolutionary & environmental sciences

For a reference copy of the document with all sections, see nature.com/documents/nr-reporting-summary-flat.pdf

Life sciences study design

All studies must disclose on these points even when the disclosure is negative.

Sample size	We derived our outcome of interest (longitudinal decline) in up to 103,938 UK Biobank participants. Cross-sectional analyses were performed in up to 405,979 UKBB individuals
Data exclusions	We removed individuals with missing data in measures of cognitive and physical aging.
Replication	We did not select an independent replication sample. There are no genotype datasets of similar size in the UK for which our outcome measure could be derived, making replication currently not feasible.
Randomization	Not applicable
Blinding	Not applicable

Reporting for specific materials, systems and methods

We require information from authors about some types of materials, experimental systems and methods used in many studies. Here, indicate whether each material, system or method listed is relevant to your study. If you are not sure if a list item applies to your research, read the appropriate section before selecting a response.

Materials & experimental systems

n/a	Involved in the study	
☒	☐	Antibodies
☒	☐	Eukaryotic cell lines
☒	☐	Palaeontology and archaeology
☒	☐	Animals and other organisms
☒	☐	Clinical data
☒	☐	Dual use research of concern
☒	☐	Plants

Methods

n/a	Involved in the study	
☒	☐	ChIP-seq
☒	☐	Flow cytometry
☒	☐	MRI-based neuroimaging

Plants

Seed stocks	Not applicable
Novel plant genotypes	Not applicable
Authentication	Not applicable
